# The STAR care pathway for patients with pain at 3 months after total knee replacement: a multicentre, pragmatic, randomised, controlled trial

**DOI:** 10.1016/S2665-9913(21)00371-4

**Published:** 2022-01-28

**Authors:** Vikki Wylde, Wendy Bertram, Emily Sanderson, Sian Noble, Nicholas Howells, Tim J Peters, Andrew D Beswick, Ashley W Blom, Andrew J Moore, Julie Bruce, David A Walsh, Christopher Eccleston, Shaun Harris, Kirsty Garfield, Simon White, Andrew Toms, Rachael Gooberman-Hill, STAR trial group, Amanda Burston, Jane Dennis, Paul Dieppe, Benjamin Burston, Vikram Desai, Tim Board, Colin Esler, Michael Parry, Jonathan R.A. Phillips

**Affiliations:** a Bristol Medical School, University of Bristol, Bristol, UK; b National Institute for Health Research Bristol Biomedical Research Centre, University Hospitals Bristol and Weston NHS Foundation Trust and the University of Bristol, UK; c North Bristol NHS Trust, Bristol, UK; d Bristol Trials Centre, University of Bristol, Bristol, UK; e Warwick Clinical Trials Unit, Division of Health Sciences, University of Warwick, Warwick, UK; f Pain Centre, Versus Arthritis/National Institute for Health Research Nottingham Biomedical Research Centre, University of Nottingham and Sherwood Forest Hospitals NHS Foundation Trust, Nottingham, UK; g Centre for Pain Research, The University of Bath, Bath, UK; h Cardiff and Vale University Health Board, Cardiff, UK; i Royal Devon and Exeter NHS Foundation Trust, Exeter, UK

## Abstract

**Background:**

Approximately 20% of people experience chronic pain after total knee replacement, but effective treatments are not available. We aimed to evaluate the clinical effectiveness and cost-effectiveness of a new care pathway for chronic pain after total knee replacement.

**Methods:**

We did an unmasked, parallel group, pragmatic, superiority, randomised, controlled trial at eight UK National Health Service (NHS) hospitals. People with chronic pain at 3 months after total knee replacement surgery were randomly assigned (2:1) to the Support and Treatment After Replacement (STAR) care pathway plus usual care, or to usual care alone. The STAR intervention aimed to identify underlying causes of chronic pain and enable onward referrals for targeted treatment through a 3-month post-surgery assessment with an extended scope practitioner and telephone follow-up over 12 months. Co-primary outcomes were self-reported pain severity and pain interference in the replaced knee, assessed with the Brief Pain Inventory (BPI) pain severity and interference scales at 12 months (scored 0–10, best to worst) and analysed on an as-randomised basis. Resource use, collected from electronic hospital records and participants, was valued with UK reference costs. Quality-adjusted life-years (QALYs) were calculated from EQ-5D-5L responses. This trial is registered with ISRCTN, ISRCTN92545361.

**Findings:**

Between Sept 6, 2016, and May 31, 2019, 363 participants were randomly assigned to receive the intervention plus usual care (n=242) or to receive usual care alone (n=121). Participants had a median age of 67 years (IQR 61 to 73), 217 (60%) of 363 were female, and 335 (92%) were White. 313 (86%) patients provided follow-up data at 12 months after randomisation (213 assigned to the intervention plus usual care and 100 assigned to usual care alone). At 12 months, the mean between-group difference in the BPI severity score was −0·65 (95% CI −1·17 to −0·13; p=0·014) and the mean between-group difference in the BPI interference score was −0·68 (−1·29 to −0·08; p=0·026), both favouring the intervention. From an NHS and personal social services perspective, the intervention was cost-effective (greater improvement with lower cost), with an incremental net monetary benefit of £1256 (95% CI 164 to 2348) at £20 000 per QALY threshold. One adverse reaction of participant distress was reported in the intervention group.

**Interpretation:**

STAR is a clinically effective and cost-effective intervention to improve pain outcomes over 1 year for people with chronic pain at 3 months after total knee replacement surgery.

**Funding:**

National Institute for Health Research.

## Introduction

Primary total knee replacement is a common surgical procedure, with more than 100 000 operations done annually in the UK.^1^ The principal aim of surgery is to reduce pain and improve function, which in 97% of patients relates to a diagnosis of osteoarthritis.^1^ However, approximately 20% of people report chronic pain after total knee replacement,^2^ with chronic pain after surgery defined as pain that occurs or increases in intensity at 3 months or longer after surgery.^3^ People with ongoing pain at 3 months after surgery are often disappointed with their outcome and struggle to make sense of their ongoing pain.^4^ Despite the high prevalence and impact of chronic pain after total knee replacement, there is little evidence available on how best to treat it.^5^

Chronic pain after total knee replacement is multifactorial, with biological, mechanical, and psychosocial contributing factors.^6^ Biological causes can include the sensitising impact of long-term pain from osteoarthritis, development of complex regional pain syndrome, inflammation, infection, and localised nerve injury. Mechanical causes can include prosthesis loosening, malalignment, and instability. Psychological factors, such as pain catastrophising and depression, can also influence outcomes. Although interventions that target each of these factors might be available, they often cannot be accessed through any single, coordinated care pathway. This highlights a need for personalised management approaches that account for these factors. Evaluations of multifaceted interventions tailored to individuals, including those that improve access to existing treatments, are needed.^7^ However, no such interventions have yet been evaluated for people with chronic pain after total knee replacement.^5^

**Research in context**

**Evidence before this study**

20% of people experience chronic pain after knee replacement surgery. Our previously published systematic reviews did not identify any multifactorial interventions for chronic pain after knee replacement or other surgeries. On Oct 27, 2021, we updated all searches with terms relating to large joint arthroplasty, post-surgical pain, and randomised controlled trials. Systematic reviews and trial registry records were checked. After screening and evaluation by two reviewers, we identified four published randomised evaluations of interventions for chronic pain after knee replacement, and eight further registered studies. No published study was at high risk of bias according to the Cochrane Risk of Bias tool. Published studies each evaluated a single intervention component: intra-articular botulinum toxin injection, neurolysis or denervation therapy, or topical lidocaine. No completed evaluations of multifaceted and personalised interventions were identified.

**Added value of this study**

This study identifies a multifaceted and personalised care pathway for chronic pain after knee replacement that is effective and cost-effective. The Support and Treatment after Replacement (STAR) intervention identifies and assesses patients with ongoing pain at 3 months after total knee replacement and directs them to the most appropriate treatment or management. We found evidence that the STAR intervention is effective in reducing the severity of chronic pain and its interference with daily activities after total knee replacement. The intervention is acceptable to patients and cost-effective. Implementation of this intervention into routine health care could provide relief from early chronic pain after knee replacement.

**Implications of all the available evidence**

The diverse possible causes of chronic pain require personalised interventions with multiple treatment and management options. The STAR intervention is, to the best of our knowledge, the first clinically effective and cost-effective intervention for chronic pain after knee replacement.

Currently, preoperative identification of individuals at high risk of chronic pain after total knee replacement to allow for targeted, preventive intervention is challenging.^5^ Therefore, the postoperative recovery period provides an important window to intervene to prevent pain chronicity. Early identification of individuals with chronic post-surgical pain is an important component of intervention, because addressing pain in a timely manner is likely to reduce the risk of long-term persistence.^7^

We aimed to evaluate the clinical effectiveness and within-trial cost-effectiveness of a novel, personalised, and multifaceted care pathway, incorporating early postoperative assessment and referral for targeted treatment compared to usual care for people with chronic pain after total knee replacement.

## Methods

### Study design and participants

We did an unmasked, parallel group, pragmatic, superiority, randomised, controlled trial with a 2:1 intervention-to-control randomisation ratio, and embedded economic evaluation and qualitative studies at eight UK National Health Service (NHS) hospitals (appendix p 36). Ethics approval was obtained from the South West-Central Bristol Research Ethics Committee (16/SW/0154). The study protocol^8^ and screening procedures^9^ have been published previously, and a CONSORT checklist is provided in the appendix (p 3). This trial is registered with ISRCTN, ISRCTN92545361.

Eligible patients were adults (ages 18 years and older) who received a primary total knee replacement because of osteoarthritis and who reported pain in their replaced knee at 3 months after surgery. Exclusion criteria included lack of capacity to provide informed consent, previous participation for the contralateral knee, and participation in another study that would interfere with the trial.

Eight high-volume NHS orthopaedic hospitals posted screening packs to consecutive patients who had undergone total knee replacement surgery 8 weeks previously. The screening pack comprised a study information booklet, consent form, and the Oxford Knee Score (OKS).^10^ The OKS is a validated 12-item joint-specific questionnaire that assesses knee pain and function. Our previous research identified that patients with a score of 14 or lower on the 7-item OKS pain component (on a scale of 0–28; worst to best) after surgery have pain that negatively affects their health-related quality of life;^11^ therefore, this cutoff was chosen as our eligibility criterion for pain. Patients who returned a consent form and who scored 14 or lower on the OKS pain component were invited to complete a second telephone OKS at 10 weeks after surgery to confirm their pain status. A recruitment consultation was then arranged for eligible, interested patients. After providing informed, written consent, participants completed a baseline questionnaire. Patient representatives collaborated on the design and conduct of the study.

### Randomisation and masking

Participants were randomly assigned to receive the Support and Treatment After Replacement (STAR) pathway and usual care or usual care alone at 12 weeks following surgery. Randomisation was done on a 2:1 basis to yield sufficient throughput to enable pragmatic assessment of the clinical effectiveness and cost-effectiveness of the intervention, and to increase the reliability and power of the per-protocol and complier average causal effect (CACE) analyses.^8^ Randomisation with assignment concealment was done by the central coordinating team by use of a computer-generated web-based system provided by the Bristol Randomised Trials Collaboration. Assignment was stratified by hospital and minimised by baseline Brief Pain Inventory (BPI) Severity and Interference Scale scores,^12^ categorised into tertiles (0 to ≤4, 4 to ≤7, and 7 to 10 for severity, and 0 to ≤4·4, 4·4 to ≤7, and 7 to 10 for interference) based on data collected from 15 patients with pain after knee replacement as part of our intervention development work.^13^ Masking of participants or staff delivering the intervention was not possible due to the nature of the intervention. Trial personnel were not masked except for assessors who collected outcome measures over the telephone from participants who did not return a questionnaire.

### Procedures

All participants received usual postoperative care as provided by their hospital.^8^ This included a routine 8-week postoperative follow-up, and some clinicians provided an additional 3-month appointment, which might have occurred before or after the participant was randomly assigned. Surgical teams followed their usual follow-up protocols, but this did not include routine assessment and follow-up by health-care professionals specialising in pain. The control group for this trial received only the usual care pathway.

The intervention was the STAR care pathway, which was developed according to UK Medical Research Council guidance on complex intervention development. Full details of the development and design of the intervention are reported elsewhere.^13^ Briefly, the process incorporated a systematic review, survey of current practice, qualitative research, patient involvement activities, consensus work with health professionals, refinement of intervention delivery, and collection of views about implementation. The intervention consisted of an assessment clinic appointment with a trained extended scope practitioner (ESP; an allied health-care professional with specialist orthopaedic training) and up to six telephone follow-up calls over 12 months. The 1 h assessment appointment was held as soon as possible after assignment (3–4 months after the surgery) and involved: clinical history; review of patient-reported outcomes, including measures of pain (Brief Pain Inventory^12^), depression (Hospital Anxiety and Depression Scale^14^), and neuropathic pain (PainDETECT^15^ and Douleur Neuropathique 4^16^); knee examination to evaluate knee tenderness, wound healing or suspicion of deeper infection (or both), range of motion, alignment, stability, patellofemoral joint function, and signs of complex regional pain syndrome; evaluation of anteroposterior weightbearing long leg alignment, lateral, and patella skyline knee radiographs for evidence of fracture or concerns with alignment, fixation, sizing, or implant position; and blood test for markers of infection (C-reactive protein).

The STAR care pathway was multifaceted and personalised according to participant needs. Based on assessment findings, participants were referred to existing NHS services for treatment targeted at potential underlying causes of pain. This could include one or more of the following referrals: to an orthopaedic surgeon for pain attributable to surgical factors or suspected infection; to a physiotherapist for muscle strengthening and exercise; to a general practitioner (GP) for further assessment and treatment of depression or anxiety (treatment choice at GP discretion); to a GP for treatment of neuropathic pain with amitripyline, gabapentin, or pregabalin for 3 months, with instructions to trial an alternative if the first medication is not tolerated, followed by recommendations for referral to pain services if needed; to a pain specialist (via GP) for treatment of complex regional pain syndrome; or other referrals as deemed necessary.

Reflecting their individual needs, participants could receive more than one referral simultaneously. All participants who attended the assessment clinic received a telephone follow-up call from an ESP, at 6 weeks after the assessment. The initial call was followed by up to five further calls over 12 months, to discuss whether existing referrals had taken place and whether any further referrals might be appropriate. Wherever possible, participants were followed up by the same ESP who had assessed their pain in clinic. Monitoring was also available if referral to none of the specialists was deemed clinically appropriate, which comprised symptom review (telephone follow-up up to five times over 12 months), with the potential for referral if clinically indicated.

All ESPs attended training sessions with a consultant knee surgeon experienced in the specialist assessment of patients with problematic total knee replacement. ESPs also received a training manual and were provided with ongoing support from the research team. The STAR intervention training manual is available online. Intervention fidelity was assessed by observation of clinic appointments and telephone follow-up.

### Outcomes

Data were collected at baseline (3 months after the surgery) and then at 6 months and 12 months after randomisation. Outcomes map onto the core outcome set for chronic pain after total knee replacement.^17^ The co-primary outcomes were self-reported pain severity and self-reported pain interference in the replaced knee, assessed with the BPI,^12^ at 12 months after randomisation. Each scale is scored 0–10 (best to worst), and a difference of one scale point is deemed the minimally important difference for both scales.^18^ Secondary outcome measures were the OKS,^10^ painDETECT,^15^ Douleur Neuropathique 4 (DN-4),^16^ Hospital Anxiety and Depression Scale (HADS),^14^ Pain Catastrophizing Scale (PCS),^19^ Possible Solutions to Pain Questionnaire (PaSol),^20^ Patient Satisfaction Scale (PSS),^21^ single-item questions on pain frequency and comparison of pain to pre-operative pain, ICEpop CAPability measure for Adults (ICECAP-A),^22^ EQ-5D-5L,^23^ Short Form-12 (SF-12),^24^ and body diagram to assess chronic widespread pain.^25^ Data on adverse reactions (adverse events directly attributable to the intervention) were collected by participant self-report in study questionnaires and monitored closely to ensure the ongoing safety of participants. The outcome for the economic evaluation was the quality-adjusted life-year (QALY). Utility values were derived from the EQ-5D-5L with the validated mapping function to the 3-level valuation set as recommended by the National Institute for Health and Care Excellence (NICE).^26^ The area under the curve approach, taking into account deaths occurring during the study, was used to calculate individual patient QALYs.

### Measurement and valuation of resource use data

A UK NHS and personal social services perspective was used for the primary cost-effectiveness analysis. This was broadened to include participants' costs for a secondary analysis. Health service use relating to chronic pain in the operated knee was recorded from randomisation to 12 months' follow-up—the time horizon of the cost-effectiveness analysis. Study proformas captured ESPs' time for the assessment and follow-up calls. Information on inpatient stays and outpatient visits at the treating hospitals was obtained from hospital electronic systems (appendix p 23). Data on primary and community-based health care, use of personal social services, and costs incurred by patients were collected by participant self-report in study questionnaires. Unit costs used to value the resource use (2019–20 UK prices) are detailed in the appendix (p 29).

After the 12-month follow-up, a purposive sample of 27 participants from the intervention group were interviewed about their experiences of the STAR care pathway. Findings of this qualitative analysis will be reported separately at a later date.

### Statistical analysis

Full details of the sample size calculation and planned analyses are provided in the protocol^8^ and statistical analysis plan (SAP).^27^ We calculated that a sample size of 285 patients would yield a power of 80% to detect a standardised difference of 0·40 SD and a power of 90% to detect a standardised difference of 0·35 SD (corresponding to 0·8 and 0·7 scale points, respectively) in the BPI between groups at 12 months after randomisation with a two-sided 5% significance level and the 2:1 randomisation ratio. Accounting for loss to follow-up of 25%, it was estimated that 381 participants (254 in the intervention group and 127 in the control group) would need to be randomly assigned to obtain primary outcome data from 285 participants.

Data analyses were done with Stata (version 15.1) and in accordance with CONSORT guidelines, commencing with descriptive analyses to compare groups at baseline. The primary comparative analysis included all available primary outcome data (ie, without imputation), with individuals in the groups to which they were randomly assigned regardless of adherence to protocol. The BPI scores at 12 months after randomisation between groups were compared with linear regression models, adjusting for the respective baseline score and minimisation or stratification variables. The secondary outcomes were analysed with regression models in a similar manner to the primary analysis.

Sensitivity analyses involved multiple imputation with chained equation techniques for missing values on the co-primary outcomes, adjusting for time between randomisation and follow-up; and restricting the primary analysis to those participants who attended the assessment clinic within 4 months of surgery. The (interim) outcome data at 6 months after randomisation were included in repeated-measures regression models for the primary outcomes, including interaction terms for group-by-time differences in effect on the outcome.^27^ A post-hoc descriptive responder analysis was done to quantify the number of patients who improved by 30% or more (considered to reflect a moderately important improvement^18^); stayed within a 30% difference (ie, worsening or improvement in pain score); and declined by 30% or more from baseline to 12 months on the BPI subscores.

Using an interaction term between treatment and subgroup variable (primarily in continuous form where available), pre-specified subgroup analyses for primary outcomes were done to investigate differential treatment effects according to hospital, OKS, and PaSol score. A post-hoc subgroup analysis was done according to completion of the primary outcomes before and after the first lockdown in the UK due to the COVID-19 pandemic (March, 2020).

Exploratory analyses were done to account for participant adherence to their allocated intervention. For the intervention group, adherence was defined as attendance at the STAR assessment clinic and was assumed for all participants in the usual care group. Although descriptive per-protocol analyses would be presented in any event, the statistical analysis plan prespecified that CACE models would only be used (to remove bias in such comparisons) if adherence to the intervention fell below 95%.^27^

### Cost-effectiveness analysis

All comparative analyses were done on an as-randomised basis and there was no discounting of costs or effects given the 1-year trial duration. A seemingly unrelated regression model, in which joint modelling of the cost and the QALY equations is used to account for the potential correlations between costs and outcomes, was used to estimate incremental costs and QALYs. Both the cost and QALY components were adjusted for baseline minimisation and stratification variables; the QALY component was also adjusted for baseline utility. The regression outputs were used to estimate mean costs and QALYs and the between-group difference in mean costs and QALYs (and their associated 95% CIs); the incremental net monetary benefit statistic, which is defined as (incremental benefit × willingness to pay threshold) – incremental cost, using the NICE willingness to pay threshold of £20 000 per QALY; and the cost-effectiveness acceptability curve at different willingness-to-pay thresholds. Details of sensitivity analyses done to address uncertainty are provided in the appendix (pp 33–35).

Multiple imputation by chained equations with predictive mean matching was used to address missing data. The imputation model, which was run by group, included baseline utility, sex, hospital, baseline BPI severity and interference scores, and baseline OKS. To maximise the complete data available for the model, at both 6 months and 12 months, utilities and costs for each resource use category were used. Rubin's rules were used to combine the 56 individual imputations.

### Role of the funding source

The funder of the study had no role in study design or trial conduct, data collection, data analysis, data interpretation, or writing of the report.

## Results

**Figure 1:**
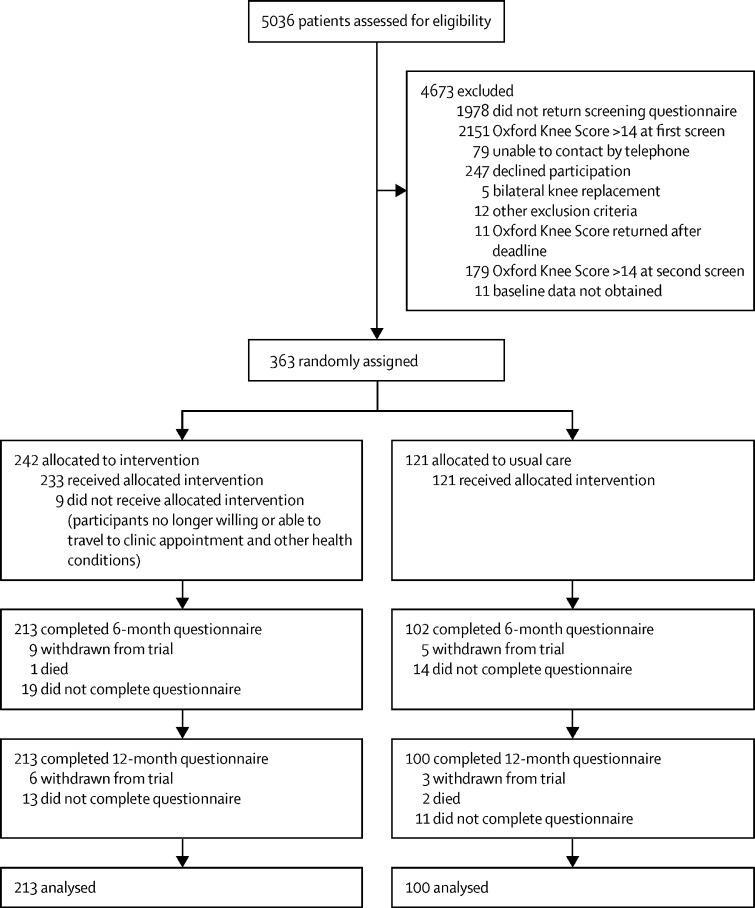
Trial profile

Between Sept 6, 2016, and May 31, 2019, screening packs were posted to 5036 patients 10 weeks after they underwent surgery. 3058 (61%) patients returned a completed screening questionnaire, of whom 907 (30%) reported pain in their replaced knee (≤14 on OKS pain component). Demographics of responders and non-responders were similar (appendix p 8). A second OKS, administered by telephone, at 12 weeks after surgery, was completed by 553 (61%) of 907 patients; 374 (41%) of 907 patients who reported pain at 10 weeks remained in pain at 12 weeks and met the trial eligibility criteria, and 363 patients were randomly assigned to receive either the intervention plus usual care (n=242) or usual care alone (n=121; figure 1). Participants' baseline characteristics are provided in table 1. There were no substantial baseline imbalances between groups. Participants had a median age of 67 years (IQR 61–73), 217 (60%) were female, 335 (92%) were White, mean unadjusted baseline BPI severity at 3 months after surgery was 5·24 (SD 1·69), and mean BPI interference was 6·28 (1·92). Given the 2:1 randomisation ratio, withdrawals from the trial were balanced between groups, with 15 participants in the intervention group and eight in the usual care group withdrawing (figure 1). Three deaths occurred during the trial: one in the intervention group and two in the usual care group; all were unrelated to the trial. 313 participants were included in the analysis of the primary outcome at 12 months after randomisation: 213 (88%) of 242 participants in the intervention group and 100 (83%) of 121 in the usual care group.

**Table 1:** Baseline characteristics of randomised participants

		**Usual care (n=121)**	**Intervention (n=242)**
Median age, years (IQR)		68 (62–74)	67 (61–73)
Sex*			
	Male	45 (37%)	101 (42%)
	Female	76 (63%)	141 (58%)
Marital status			
	Single	11 (9%)	14 (6%)
	Married/partner	81 (67%)	170 (70%)
	Separated	13 (11%)	22 (9%)
	Widowed	11 (9%)	34 (14%)
	Missing	5 (4%)	2 (1%)
Living arrangement			
	Alone	22 (18%)	56 (23%)
	With partner	82 (68%)	171 (71%)
	With somebody else	11 (9%)	11 (5%)
	Other	1 (1%)	2 (1%)
	Missing	5 (4%)	2 (1%)
Ethnic group			
	White	109 (90%)	226 (93%)
	Mixed	0	1 (<1%)
	Asian	5 (4%)	6 (2%)
	Black	1 (1%)	4 (2%)
	Other	1 (1%)	3 (1%)
	Missing	5 (4%)	2 (1%)
Education level			
	Left before age 16 years	8 (7%)	14 (6%)
	Left at age 16 years	61 (50%)	133 (55%)
	College	22 (18%)	39 (16%)
	University degree	2 (2%)	13 (5%)
	Post-graduate degree	12 (10%)	12 (5%)
	Other	0	3 (1%)
	Missing	16 (13%)	28 (12%)

Data are n (%), unless otherwise stated.

* Collected by self-report.

**Figure 2:**
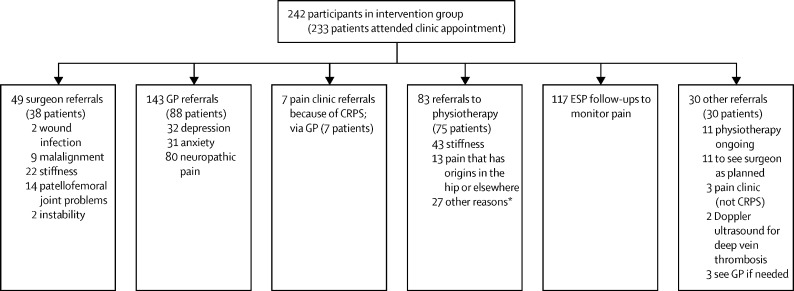
Referrals made for patients in the intervention group CRPS=complex regional pain syndrome. ESP=extended scope practitioner. GP=general practitioner. *Ongoing or additional physiotherapy (n=8); hydrotherapy (n=4); weakness or strengthening (n=12); and no reason specified (n=3).

Of those randomly assigned to the STAR care pathway, 233 (96%) of 242 attended the clinic appointment. Reasons for non-attendance included participants no longer being willing or able to travel to hospital appointments and other health conditions. Overall, 172 (71%) of 242 patients attended the appointment by 4 months after surgery. Participants had a median of two (IQR 1–2) onward treatment referrals (range 1–9) across a range of disciplines (figure 2). 97 (42%) of 233 patients received one referral; 78 (33%) received two referrals; 36 (15%) received three referrals; 14 (6%) received four referrals; four (2%) received five referrals; three (1%) received seven referrals; and one (<1%) received nine referrals. Assessment of the fidelity of the intervention identified one instance of non-compliance at a site where referrals were not being made for neuropathic pain and depression or anxiety. Corrective action was taken through discussion and training with the local research team; preventive actions included additional training for all sites and monthly review of assessment documents for compliance.

The primary analysis yielded an adjusted mean between-group difference in the BPI severity score at 12 months after randomisation that favoured the STAR care pathway (−0·65 [95% CI −1·17 to −0·13]; p=0·014; table 2). The adjusted mean between-group difference in the BPI interference score at 12 months also favoured the STAR care pathway (−0·68 [95% CI −1·29 to −0·08]; p=0·026; table 2). Similar results were observed in the per-protocol analyses (the high level of adherence meant that CACE analyses were not required), and those involving multiple imputation of missing outcome data, adjustment for time to follow-up, and exclusion of participants involved in a similar interventional trial (appendix pp 14–21). Further analyses involving assumptions about missing data indicated that it would take an extreme assumption (for instance, assuming a best case value of zero for all missing data in both groups) for the evidence of a between-group difference to be attenuated. In the post-hoc descriptive responder analysis, 132 (62%) of 212 participants in the intervention group, and 54 (54%) of 100 in the usual care group displayed an improvement in pain severity of 30% or greater by 12 months. For pain interference, 135 (63%) of 213 participants improved in the intervention group, as did 59 (59%) of 100 in the usual care group (appendix p 22).

**Table 2:** Primary and secondary outcomes at 12 months (continuous outcomes)

	**Usual care group**		**Intervention group**		Mean difference (95% CI)*	**p value**
	Number of patients	Mean (SD)	Number of patients	Mean (SD)		
**Co-primary outcomes**						
BPI severity (0 to 10, best to worst)	100	3·67 (2·52)	212	3·14 (2·43)	−0·65 (−1·17 to −0·13)	0·014
BPI interference (0 to 10, best to worst)	100	4·08 (2·90)	213	3·52 (2·79)	−0·68 (−1·29 to −0·08)	0·026
**Secondary outcomes**						
OKS (0 to 48, worst to best)	93	27·16 (9·72)	201	28·44 (10·23)	2·68 (0·58 to 4·78)	0·013
DN-4 (0 to 7, best to worst)	94	2·94 (2·12)	195	3·07 (2·07)	−0·10 (−0·55 to 0·35)	0·653
PainDETECT (−1 to 38, best to worst)	94	12·69 (7·53)	198	12·87 (7·70)	−0·93 (−2·51 to 0·65)	0·249
Pain Catastrophizing Scale† (0 to 52, best to worse)	91	13·75 (13·07)	195	11·82 (12·60)	0·90 (0·70 to 1·16)	0·428
PaSol: solving pain (0 to 24, worst to best)	89	13·21 (7·93)	193	12·69 (8·11)	−1·18 (−3·09 to 0·74)	0·226
PaSol: meaningful life (0 to 30, worst to best)	90	19·81 (8·28)	193	19·20 (8·92)	−0·64 (−2·83 to 1·55)	0·565
PaSol: acceptance of pain (0 to 18, worst to best)	87	8·55 (5·32)	191	8·54 (5·85)	−0·22 (−1·65 to 1·20)	0·757
PaSol: belief in solution (0 to 12, worst to best)	90	5·91 (4·34)	193	5·74 (4·34)	−0·38 (−1·47 to 0·70)	0·490
Patient Satisfaction Scale (25 to 100, worst to best)	89	68·73 (21·71)	197	71·10 (23·46)	3·79 (−1·47 to 9·06)	0·157
ICECAP-A (−0·001 to 1, worst to best)	91	0·78 (0·21)	197	0·78 (0·19)	0·03 (−0·004 to 0·06)	0·085
SF-12 (physical; 0 to 100, worst to best)	93	36·96 (9·95)	195	37·70 (9·71)	2·07 (−0·10 to 4·23)	0·061
SF-12 (mental; 0 to 100, worst to best)	93	48·58 (11·25)	195	48·07 (11·04)	−0·08 (−2·29 to 2·12)	0·940
HADS: anxiety (0 to 21, best to worst)	92	5·96 (4·67)	198	6·05 (4·37)	−0·70 (−1·47 to 0·08)	0·079
HADS: depression (0 to 21, best to worst)	90	6·07 (4·27)	197	5·98 (3·84)	−0·69 (−1·47 to 0·10)	0·086

BPI=Brief Pain Inventory. OKS=Oxford Knee Score. DN-4=Douleur Neuropathique 4. PaSol=Pain Solutions Questionnaire. ICECAP-A=ICEpop CAPability measure for Adults. SF-12=12-item Short Form Survey. HADS=Hospital Anxiety and Depression Scale.

* All analyses are adjusted for hospital site, baseline BPI subscores and baseline score for each outcome measure.

† Analysed on a log scale because this improved the normality of the residuals. Estimates are back-transformed to the original scale.

At 6 months after randomisation, the mean BPI severity score favoured the intervention group (mean between-group difference −0·55 [95% CI −1·05 to −0·06]; p=0·028), with similar results for the BPI interference score (mean between-group difference −0·71 [–1·28 to −0·15]; p=0·014). Repeated-measures analysis showed no evidence of a difference in treatment effect at 6 months compared with 12 months, consistent with an effect at 6 months that is maintained at 12 months after randomisation for both the BPI pain and interference subscales (appendix p 15). The OKS was better with the intervention than with usual care at 12 months (mean between-group difference 2·68 [95% CI 0·58 to 4·78]; p=0·013), whereas other secondary outcome measures did not reveal additional treatment effects (Table 2, Table 3).

**Table 3:** Secondary outcomes at 12 months (categorical outcomes)

	**Usual care group**	**Intervention group**	Odds ratio*(95% CI)	**p value**
**Chronic widespread pain**				
Yes	7/100 (7%)	9/212 (4%)	0·61 (0·20 to 1·91)	0·399
No	93/100 (93%)	203/212 (96%)	..	..
**Pain frequency in past 24 h**				
Rarely	21/100 (21%)	55/213 (26%)	0·64 (0·34 to 1·21)	0·170
Sometimes	28/100 (28%)	63/213 (30%)	..	..
Often	20/100 (20%)	41/213 (19%)	..	..
Most of the time	18/100 (18%)	35/213 (16%)	..	..
All of the time	13/100 (13%)	19/213 (9%)	..	..
**Pain frequency in past 4 weeks**				
Rarely	16/93 (17%)	45/198 (23%)	0·55 (0·27 to 1·11)	0·095
Sometimes	26/93 (28%)	50/198 (25%)	..	..
Often	22/93 (24%)	43/198 (22%)	..	..
Most of the time	20/93 (22%)	42/198 (21%)	..	..
All of the time	9/93 (10%)	18/198 (9%)	..	..
**Comparison of pain**†				
Much better	44/91 (48%)	111/198 (56%)	0·62 (0·34 to 1·12)	0·113
A bit better	21/91 (23%)	39/198 (20%)	..	..
The same	11/91 (12%)	15/198 (8%)	..	..
A bit worse	8/91 (9%)	19/198 (10%)	..	..
Much worse	7/91 (8%)	14/198 (7%)	..	..

Data are number of patients in the format n/N (%), unless otherwise stated. All descriptive statistics are unadjusted. BPI=Brief Pain Inventory.

* All analyses are adjusted for hospital site, baseline BPI subscores and baseline score for each outcome measure.

† Comparison of pain: how does the pain you have in your replaced knee now compare to the pain you had in your knee before your operation?

Planned subgroup analyses showed evidence of a differential effect according to a continuous measure of the baseline OKS on BPI severity (p_interaction_=0·022) and BPI interference (p_interaction_=0·002), with larger treatment effects among patients with a worse baseline OKS at 3 months after surgery. Differential treatment effects were not identified when baseline OKS was treated as a categorical variable, and nor for the other subgroup variables—trial site, baseline PaSol, and co-primary outcomes obtained before or after the first lockdown in the UK from March to June, 2020, due to the COVID-19 pandemic (appendix pp 15–17).

Complete data on NHS and personal social services costs were available for 230 (63%) of 363 participants, of whom 160 (70%) also had data available on patient costs. The cost-effectiveness analysis was therefore done on a multiple imputed dataset of all 363 participants. Mean resource use was similar between the two groups for most categories (appendix pp 30–32). There was, however, a greater mean number of inpatient stays in the usual care group than in the intervention group (0·25 *vs* 0·13 inpatient stays). The usual care group also had a slightly higher mean number of home changes and equipment provided by the NHS or personal social services at both 6 months (0·40 *vs* 0·38 home changes or pieces of equipment) and 12 months (0·10 *vs* 0·05), and a greater number of hours of unpaid leave at both 6 months (12·78 *vs* 7·06 h) and 12 months (8·11 *vs* 2·61 h). There was a greater mean number of GP contacts at both 6 months (0·97 *vs* 0·51) and 12 months (0·51 *vs* 0·32) in the intervention group compared with the usual care group. The cost-effectiveness analysis from the NHS and personal social services perspective showed that costs in the intervention group were £724 (95% CI −1500 to 51) less than the usual care group and there were an additional 0·03 (95% CI −0·008 to 0·06) QALYs with the intervention compared with usual care (table 4). This meant the incremental net monetary benefit at £20 000 per QALY threshold was £1256 (95% CI 164 to 2348), indicating a 98·79% probability that the intervention is the cost-effective option when compared with usual care (appendix p 28). Similarly, from a patient perspective, and all perspectives combined, the intervention group remained dominant. The greater number of hospital admissions (from the NHS and personal social services perspective), and the greater number of hours of unpaid leave (from the patient perspective in the usual care group) were the cost drivers of these results. Sensitivity analyses confirmed these results, in that the intervention group was dominant and the probability that the intervention is the cost-effective option did not go below 96·5% for any of these analyses (appendix p 33).

**Table 4:** Cost-effectiveness results

	**Number of patients**	Mean adjusted costs*(95% CI)	Mean adjusted QALYs*(95% CI)	**Mean incremental costs (95% CI)**	**Incremental QALYs (95% CI)**	**Mean incremental net monetary benefit at £20 000 per QALY (95% CI)**
**NHS and personal social services perspective**						
Intervention	242	£1961·84 (1531·05 to 2392·63)	0·52 (0·50 to 0·54)	..	..	..
Usual care	121	£2686·28 (2034·86 to 3337·69)	0·50 (0·47 to 0·52)	..	..	..
Intervention *vs* usual care	..	..	..	−£724·43 (−1500·27 to 51·39)	0·027 (−0·008 to 0·06)	£1256 (164 to 2348)
**Patient perspective**						
Intervention	242	£375·52 (155·32 to 595·71)	0·52 (0·50 to 0·54)	..	..	..
Usual care	121	£682·90 (307·25 to 1058·55)	0·50 (0·47 to 0·53)	..	..	..
Intervention *vs* usual care	..	..	..	−£307·38 (−745·70 to 130·94)	0·027 (−0·008 to 0·06)	£865 (−91 to 1822)
**NHS, personal social services, and patient perspective**						
Intervention	242	£2337·36 (1850·53 to 2824·19)	0·52 (0·50 to 0·54)	..	..	..
Usual care	121	£3369·17 (2603·26 to 4135·09)	0·50 (0·47 to 0·52)	..	..	..
Intervention *vs* usual care	..	..	..	−£1031·81 (−1936·58 to −127·05)	0·027 (−0·008 to 0·06)	£1564 (369 to 2758)

QALY=quality-adjusted life-year. NHS=UK National Health Service. BPI=Brief Pain Inventory.

* All variables are adjusted for hospital site and baseline BPI subscores; additionally, QALYs were adjusted for baseline utility.

One adverse reaction was reported on Feb 5, 2018, in which there was a report of participant distress in the intervention group. This was related to a protocol deviation in which the ESP had referred the participant to their GP for anxiety and depression but had not discussed the referral with the participant first. Corrective and preventive actions were taken, and no further adverse reactions were reported.

## Discussion

Our findings indicate that the STAR care pathway is a clinically effective and cost-effective intervention for reducing pain severity and interference over a 1-year period for people with pain at 3 months after total knee replacement surgery. The intervention was designed to provide people who have chronic pain after total knee replacement with personalised care through a multifaceted treatment approach. The intervention addresses the need for clear pathways and referral processes to facilitate patients' access to targeted care matched to their individual pain characteristics.^7^ This model of care integrates aspects of optimal care for people with chronic pain into a post-surgical orthopaedic context. Our trial contributes to the existing evidence by providing the first robust evaluation of a multifaceted and personalised intervention for individuals with pain at 3 months after total knee replacement.

Strengths of this trial include the pragmatic design and inclusion of multiple hospitals, increasing the relevance and generalisability of the findings. We used a robust and standardised approach for early identification of patients with pain. The demographics of the trial sample, in terms of ethnicity (92% White), median age (67 years), and sex (60% female), broadly reflects the national population of individuals undergoing total knee replacement at the time of the study (95% White, mean age 69 years, 57% female).^1^, ^28^ Our choice of outcomes comprised items from a core outcome set,^17^ ensuring that the priorities of patients and health-care professionals are reflected in the findings. The intervention was developed and refined through a comprehensive series of studies and patient involvement activities,^13^ and patient adherence to the intervention was high, with 96% of participants in the intervention group attending the STAR clinic. However, 29% of patients attended clinic 4 months or more after surgery, highlighting potential challenges in care delivery within the target timeframes. A limitation of our study was that the postal screening process might have missed people with pain, as nearly half of patients did not return the screening questionnaire. Given the pragmatic nature of trial processes, this reflects the likely uptake of the intervention if implemented into routine care. Another potential weakness of the trial was that it was not possible to mask participants to treatment allocation due to the nature of the intervention, which could potentially have contributed to an overestimation of the treatment effect. There were missing outcome data, but our sensitivity analyses involving the use of multiple imputation indicated that these missing data did not substantially affect the results. Missing questionnaire data were also an issue for the cost-effectiveness analysis, which meant it was not appropriate to conduct a complete case analysis. However, the main cost driver was related to hospital admissions, for which we had 93% complete data.

The purpose of the STAR intervention is to identify people with pain early in the postoperative pathway and ensure they have timely access to targeted care and support to optimise their outcomes. Given the complexity of the intervention, there are multiple potential mechanisms that might explain how the intervention reduced pain severity and interference. This includes robust early screening processes to aid appropriate identification of patients in need of the intervention, comprehensive assessment to identify causes of pain with validated screening tools and best-practice clinical examination, personalised referrals to evidence-based treatments, continuity of care, and follow-up. Early intervention is also a key component. Chronic pain can be difficult to treat once established, because of the complex underlying mechanisms involving interactions between biological, cognitive, emotional, social, and environmental factors.^7^ STAR was designed to intervene at the stage in which acute pain can transition to chronic pain.^13^ By screening patients at 8–10 weeks post-surgery, it was possible to offer an assessment appointment to most patients within 4 months of surgery, with the aim of preventing longer-term chronicity; however, it is acknowledged that 8 weeks is early in the pain recovery trajectory and some patients' pain outcomes improved before randomisation. Another possible mechanism by which the STAR care pathway improved pain outcomes is that it provided an opportunity for patients and ESPs to engage in ongoing follow-up. Patients with pain after total knee replacement have described a sense of abandonment after surgery because of lack of follow-up and reluctance by health-care professionals to acknowledge ongoing pain.^4^ Having a longer appointment and additional follow-up with the same clinician might have provided the environment for a more caring and compassionate clinical encounter, which can improve overall wellbeing.^29^

Further research is ongoing to evaluate the longer-term effects of the STAR care pathway on outcomes and health-care use at 4 years after randomisation. We also anticipate that advances in treatment and management strategies for chronic pain will be incorporated into the STAR care pathway. The STAR intervention provides a model for care delivery that requires referrals for treatments to be guided by high-quality evidence to ensure that patients receive effective care. Therefore, future refinements of individual components of the STAR care pathway would be expected as new evidence emerges on effective interventions. Future research could also evaluate the potential transferability of this model of care to different health-care systems and other orthopaedic and non-orthopaedic elective surgeries.

In conclusion, the STAR care pathway is a clinically effective and cost-effective intervention for reducing pain severity and interference for people with pain at 3 months after total knee replacement. The potential impact of this intervention on patient care is substantial as there is a need for evidence-based treatments for patients with chronic pain after total knee replacement.^5^ Implementation of the STAR intervention into routine health-care service provision would provide patients with access to a safe, acceptable, and cost-effective treatment to improve pain outcomes.

## Data sharing

The data sets generated during the present study will be available in the University of Bristol Research Data Repository. Data will be available within 6 months following publication. Access to the data will be restricted to ensure that data are only made available to bona fide researchers for ethically approved research projects, on the understanding that confidentiality will be maintained and after a data access agreement has been signed by an institutional signatory.

## Declaration of interests

AWB and VW have received funding from Stryker for research unrelated to this work. DAW has received grant funding from Pfizer; consulting fees from Pfizer, AbbVie, GlaxoSmithKline, Reckitt- Benckiser, Galapagos, and EliLilly; and payment for lectures, presentations, and educational events from Pfizer and AbbVie, all unrelated to this work. All other authors declare no competing interests.
