# Correction to *Lancet Rheumatol* 2022; 4: e188–97

**DOI:** 10.1016/S2665-9913(23)00130-3

**Published:** 2023-05-08

*Wylde V, Bertram W, Sanderson E, et al. The STAR care pathway for patients with pain at 3 months after total knee replacement: a multicentre, pragmatic, randomised, controlled trial.* Lancet Rheumatol 2022; ***4:** e188–97*—In this Article, figure 1 has been amended to clarify that five participants allocated to the intervention and four participants allocated to usual care withdrew from the trial at 12 months. The eighth sentence of the first paragraph of the Results section has also been amended as follows: “Given the 2:1 randomisation ratio, withdrawals from the trial were balanced between groups, with 14 participants in the intervention group and nine in the usual care group withdrawing (figure 1).” These corrections have been made as of May 8, 2023.

