# Supplementary material for: The STAR care pathway for patients with pain at 3 months after total knee replacement: a multicentre, pragmatic, randomised, controlled trial
Source: Lancet Rheumatol. 2022 Jan 28;4(3):e188–97. doi: 10.1016/S2665-9913(21)00371-4 (PMC8873030; doi:10.1016/S2665-9913(21)00371-4)
Supplement: Supplementary appendix [file mmc1.pdf]

THE LANCET
Rheumatology

Supplementary appendix

This appendix formed part of the original submission and has been peer reviewed. We post it as supplied by the authors.

Supplement to: Wylde V, Bertram W, Sanderson E, et al. The STAR care pathway for patients with pain at 3 months after total knee replacement: a multicentre, pragmatic, randomised, controlled trial. *Lancet Rheumatol* 2022; published online Jan 27. https://doi.org/10.1016/S2665-9913(21)00371-4.

Appendix

Table of contents

Table 1 CONSORT checklist of information to include when reporting a randomised trial 3
Table 2: Baseline characteristics 5
Table 3: Demographics and patients who did and did not complete screening 8
Table 4: Baseline questionnaire completion by group 8
Table 5: 6 month questionnaire completion by group 8
Table 6: 12 month questionnaire completion by group 9
Table 7: Withdrawal summary by group 9
Table 8: Reasons for withdrawal 9
Table 9: Number of follow up calls received 10
Table 10: Timings of follow up calls received 10
Table 11: Protocol deviations 10
Table 12: Summary statistics at 6 months and 12 months 10
Table 13: Secondary analysis – adjusting for 'time to follow up from recruitment" 14
Table 14:Sensitivity analysis for missing data 14
Table 15: Sensitivity analysis for missing data 14
Table 16: Overlap sensitivity analysis for BPI Severity scale 14
Table 17: Sensitivity analysis for missing data 14
Table 18: Sensitivity analysis for missing data 15
Table 19: Sensitivity analysis excluding patients who had their assessment after 4 months post-operative 15
Table 20: Repeated measures analysis of BPI subscales 15
Table 21: Evidence of interaction 15
Table 22: Subgroup analysis of OKS categories 17
Table 23: Accounting for potential impact of COVID-19: Psychological outcomes 17
Table 24: OKS sensitivity analysis: missing data 17
Table 25: OKS sensitivity analysis: per protocol 18
Table 26: DN-4 sensitivity analysis: missing data 18
Table 27: DN-4 sensitivity analysis: per protocol 18
Table 28: PainDETECT sensitivity analysis: missing data 18
Table 29: PainDETECT sensitivity analysis: per protocol 18
Table 30: PCS sensitivity analysis: missing data 18
Table 31: PCS sensitivity analysis: per protocol 18
Table 32: PaSol sensitivity analysis: missing data 19
Table 33: PaSol sensitivity analysis: per protocol 19
Table 34: Patient satisfaction sensitivity analysis: missing data 19
Table 35: Patient satisfaction sensitivity analysis: per protocol 19
Table 36: ICECAP-A sensitivity analysis: missing data 19
Table 37: ICECAP-A sensitivity analysis: per protocol 19

Table 38: SF-12 sensitivity analysis: missing data ... 20
Table 39: SF-12 sensitivity analysis: per protocol ... 20
Table 40: HADS anxiety scale sensitivity analysis: missing data ... 20
Table 41: HADS anxiety scale sensitivity analysis: per protocol ... 20
Table 42: HADS depression scale sensitivity analysis: missing data ... 20
Table 43: HADS depression scale sensitivity analysis: per protocol ... 20
Table 44: Chronic widespread pain sensitivity analysis: missing data ... 20
Table 45: Chronic widespread pain sensitivity analysis: per protocol ... 21
Table 46: Frequency of pain sensitivity analysis: missing data ... 21
Table 47: Frequency of pain sensitivity analysis: per protocol ... 21
Table 48: Comparison of pain to pre-operative pain sensitivity analysis: missing data ... 21
Table 49: Comparison of pain to pre-operative pain sensitivity analysis: per protocol ... 21
Table 50: Adverse reactions ... 21
Table 51: Responder analysis ... 22
Table 52 Studies from systematic review ... 22
Table 53 CHEERS checklist ... 24
Table 54 Resources collected and their valuation (2019/20 prices excluding VAT) ... 29
Table 55 Mean resource use from an NHS and PSS perspective by category and randomised allocation ... 30
Table 56 Mean resource use from a patient perspective by category and randomised allocation[1] ... 31
Table 57 Mean costs from an NHS and PSS perspective by category and randomised allocation ... 31
Table 58 Mean utilities over time and by randomised allocation ... 32
Table 59 VAS scores over time and by randomised allocation ... 32
Table 60 One-way sensitivity analyses for the economic evaluation ... 33

Figure 1: Unadjusted mean BPI severity score with confidence intervals at baseline and follow-up ... 13
Figure 2: Unadjusted mean BPI interference score with confidence intervals at baseline and follow-up ... 13
Figure 3: Treatment effect on BPI severity over differing levels of OKS (continuous) at baseline ... 16
Figure 4: Treatment effect on BPI interference over differing levels of OKS (continuous) at baseline ... 16
Figure 5: Cost-effectiveness acceptability curve from the NHS/PSS perspective ... 28

Supplementary Information relating to the economic evaluation ... 23
STAR trial sites ... 36
Trial Steering Committee members ... 36
Members of the STAR trial group ... 36
References ... 37

Table 1 CONSORT checklist of information to include when reporting a randomised trial

Section/Topic	Item No	Checklist item	Where reported
**Title and abstract**			
	1a	Identification as a randomised trial in the title	Title
	1b	Structured summary of trial design, methods, results, and conclusions (for specific guidance see CONSORT for abstracts)	Abstract
**Introduction**			
Background and objectives	2a	Scientific background and explanation of rationale	Background
	2b	Specific objectives or hypotheses	Background
**Methods**			
Trial design	3a	Description of trial design (such as parallel, factorial) including allocation ratio	Methods – study design
	3b	Important changes to methods after trial commencement (such as eligibility criteria), with reasons	N/A
Participants	4a	Eligibility criteria for participants	Methods – participants
	4b	Settings and locations where the data were collected	Methods – study design
Interventions	5	The interventions for each group with sufficient details to allow replication, including how and when they were actually administered	Methods – procedures
Outcomes	6a	Completely defined pre-specified primary and secondary outcome measures, including how and when they were assessed	Methods – outcomes
	6b	Any changes to trial outcomes after the trial commenced, with reasons	N/A
Sample size	7a	How sample size was determined	Methods – statistical analysis
	7b	When applicable, explanation of any interim analyses and stopping guidelines	N/A
Randomisation:			
Sequence generation	8a	Method used to generate the random allocation sequence	Methods – randomisation and masking
	8b	Type of randomisation; details of any restriction (such as blocking and block size)	Methods – randomisation and masking
Allocation concealment mechanism	9	Mechanism used to implement the random allocation sequence (such as sequentially numbered containers), describing any steps taken to conceal the sequence until interventions were assigned	Methods – randomisation and masking
Implementation	10	Who generated the random allocation sequence, who enrolled participants, and who assigned participants to interventions	Methods – randomisation and masking
Blinding	11a	If done, who was blinded after assignment to interventions (for example, participants, care providers, those assessing outcomes) and how	Methods – randomisation and masking
	11b	If relevant, description of the similarity of interventions	Methods – procedures
Statistical methods	12a	Statistical methods used to compare groups for primary and secondary outcomes	Methods – statistical analysis
	12b	Methods for additional analyses, such as subgroup analyses and adjusted analyses	Methods – statistical analysis
**Results**			

Participant flow (a diagram is strongly recommended)	13a	For each group, the numbers of participants who were randomly assigned, received intended treatment, and were analysed for the primary outcome	Results – first and second paragraph; Figure 1
	13b	For each group, losses and exclusions after randomisation, together with reasons	Results – second paragraph; Figure 1
Recruitment	14a	Dates defining the periods of recruitment and follow-up	Results – first paragraph
	14b	Why the trial ended or was stopped	N/A
Baseline data	15	A table showing baseline demographic and clinical characteristics for each group	Table 1
Numbers analysed	16	For each group, number of participants (denominator) included in each analysis and whether the analysis was by original assigned groups	Tables
Outcomes and estimation	17a	For each primary and secondary outcome, results for each group, and the estimated effect size and its precision (such as 95% confidence interval)	Table 2
	17b	For binary outcomes, presentation of both absolute and relative effect sizes is recommended	Table 2
Ancillary analyses	18	Results of any other analyses performed, including subgroup analyses and adjusted analyses, distinguishing pre-specified from exploratory	Supplementary material
Harms	19	All important harms or unintended effects in each group (for specific guidance see CONSORT for harms)	Supplementary material
**Discussion**			
Limitations	20	Trial limitations, addressing sources of potential bias, imprecision, and, if relevant, multiplicity of analyses	Discussion, paragraph 2
Generalisability	21	Generalisability (external validity, applicability) of the trial findings	Discussion, paragraph 2
Interpretation	22	Interpretation consistent with results, balancing benefits and harms, and considering other relevant evidence	Discussion
**Other information**			
Registration	23	Registration number and name of trial registry	Abstract
Protocol	24	Where the full trial protocol can be accessed, if available	Methods – study design
Funding	25	Sources of funding and other support (such as supply of drugs), role of funders	Abstract, funding acknowledgement

Table 2: Baseline characteristics

		Overall	Intervention	Usual care
**Gender (male)**	**N (%)**	146 (40.22)	101 (41.74)	45 (37.19)
**Age**	**N**	363	242	120
	**Mean (SD)**	67.15 (8.71)	67.01 (8.52)	67.38 (9.14)
**Marital Status**	**N**	356	240	116
	**Single (%)**	25 (7.02)	14 (5.83%)	11 (9.48%)
	**Married/ partner (%)**	251 (70.51)	170 (70.83%)	81 (69.83%)
	**Separated (%)**	35 (9.83)	22 (9.17%)	13 (11.21%)
	**Widowed (%)**	45 (12.64)	34 (14.17%)	11 (9.48%)
**Living arrangement**	**N**	356	240	116
	**Alone (%)**	78 (21.91)	56 (23.33%)	22 (18.97%)
	**With Partner (%)**	253 (71.07)	171 (71.25%)	82 (70.69%)
	**With Somebody else (%)**	22 (6.18)	11 (4.58%)	11 (9.48%)
	**Other (%)**	3 (0.84)	2 (0.83%)	1 (0.86%)
**Ethnic Group**	**N**	356	240	116
	**White (%)**	335 (94.10)	226 (94.17%)	109 (93.97%)
	**Mixed (%)**	1 (0.28)	1 (0.42%)	0
	**Asian (%)**	11 (3.09)	6 (2.50%)	5 (4.31%)
	**Black (%)**	5 (1.40)	4 (1.67%)	1 (0.86%)
	**Other (%)**	4 (1.12)	3 (1.25%) ["Iranian", "Not sure", not specified]	1 (0.86%) ["Burmese"]
**Education level**	**N**	319	214	105
	**Before 16**	22 (6.90)	14 (6.54%)	8 (7.62%)
	**At 16**	194 (60.82)	133 (62.15%)	61 (58.10%)
	**college**	61 (19.12)	39 (18.22%)	22 (20.95%)
	**university degree**	15 (4.70)	13 (6.07%)	2 (1.90%)
	**Post-graduate**	24 (7.52)	12 (5.61%)	12 (11.43%)
	**Other**	3 (0.94)	3 (1.40%)	0
**DN-4 (neuropathic pain)**	**N**	359	240	119
	**Yes; N (%)**	267 (74.37)	181 (75.42%)	86 (72.27%)
	**No; N (%)**	92 (25.63)	59 (24.58%)	33 (27.73%)
**PainDETECT (neuropathic pain)**	**N**	363	242	121
	**Unlikely; N (%)**	76 (20.94)	45 (18.60%)	31 (25.62%)
	**Ambiguous; N (%)**	96 (26.45)	66 (27.27%)	30 (24.79%)
	**Likely; N (%)**	191 (52.62)	131 (54.13%)	60 (49.59%)
**HADS: Anxiety**	**N**	363	242	121
	**Number of "Normal" (%)**	197 (54.27)	124 (51.24%)	73 (60.33%)
	**# "Borderline" (%)**	71 (19.56)	51 (21.07%)	20 (16.53%)
	**# "Clinical" (%)**	95 (26.17)	67 (27.69%)	28 (23.14%)
**HADS: Depression**	**N**	362	242	120
	**Number of "Normal" (%)**	177 (48.90)	114 (47.11%)	63 (52.50%)
	**# "Borderline" (%)**	91 (25.14)	62 (25.62%)	29 (24.17%)
	**# "Clinical" (%)**	94 (25.97)	66 (27.27%)	28 (23.33%)

**Frequency of pain Q1**[2]	**N**	361	242	119
	**# "Rarely" (%)**	1 (0.28)	1 (0.41%)	0
	**# "Sometimes" (%)**	40 (11.08)	26 (10.74%)	14 (11.76%)
	**# "Often" (%)**	98 (27.15)	60 (24.79%)	38 (31.93 %)
	**# "Most of the time" (%)**	164 (45.43)	109 (45.04%)	55 (46.22%)
	**# "All of the time" (%)**	58 (16.07)	46 (19.01%)	12 (10.08%)
**Frequency of pain Q2**[3]	**N**	362	242	120
	**# "Rarely" (%)**	0	0	0
	**# "Sometimes" (%)**	14 (3.87)	8 (3.31%)	6 (5.00%)
	**# "Often" (%)**	102 (28.18)	68 (28.10%)	34 (28.33%)
	**# "Most of the time" (%)**	156 (43.09)	99 (40.91%)	57 (47.50%)
	**# "All of the time" (%)**	90 (24.86)	67 (27.69%)	23 (19.17%)
**How satisfied are you with the results of your surgery**	**N**	360	241	119
	**Very dissatisfied n (%)**	21(5.83)	14 (5.81%)	7 (5.88%)
	**Somewhat dissatisfied n (%)**	73 (20.28)	53 (21.99%)	20 (16.81%)
	**Somewhat satisfied n (%)**	154 (42.78)	95 (39.42%)	59 (49.58%)
	**Very satisfied n (%)**	112 (31.11)	79 (32.78%)	33 (27.73%)
**How satisfied are you with the results of your surgery for improving your pain**	**N**	360	241	119
	**Very dissatisfied n (%)**	47 (13.06)	35 (14.52%)	12 (10.08%)
	**Somewhat dissatisfied n (%)**	118 (32.78)	77 (31.95%)	41 (34.45%)
	**Somewhat satisfied n (%)**	139 (38.61)	90 (37.34%)	49 (41.18%)
	**Very satisfied n (%)**	56 (15.56)	39 (16.18%)	17 (14.29%)
**How satisfied are you with the results of your surgery for improving your ability to do housework or gardening**	**N**	359	241	118
	**Very dissatisfied n (%)**	65 (18.11)	44 (18.26%)	21 (17.80%)
	**Somewhat dissatisfied n (%)**	152 (42.34)	99 (41.08%)	53 (44.92%)
	**Somewhat satisfied n (%)**	112 (31.20)	77 (31.95%)	35 (29.66%)
	**Very satisfied n (%)**	30 (8.36)	21 (8.71%)	9 (7.63%)
**How satisfied are you with the results of your surgery for improving your ability to do leisure activities**	**N**	360	242	118
	**Very dissatisfied n (%)**	86 (23.89)	56 (23.14%)	30 (25.42%)
	**Somewhat dissatisfied n (%)**	140 (38.89)	94 (38.84%)	46 (38.98%)
	**Somewhat satisfied n (%)**	107 (29.72)	72 (29.75%)	35 (29.66%)
	**Very satisfied n (%)**	27 (7.50)	20 (8.26%)	7 (5.93%)
**Comparison of pain**[4]	**N**	362	242	120
	**# "Much Better" (%)**	79 (21.82)	51 (21.07%)	28 (23.33%)
	**# "A bit better" (%)**	70 (19.34)	47 (19.42%)	23 (19.17%)
	**# "The same" (%)**	54 (14.92)	37 (15.29%)	17 (14.17%)
	**# "A bit worse" (%)**	77 (21.27)	54 (22.31%)	23 (19.17%)
	**# "Much worse" (%)**	82 (22.65)	53 (21.90%)	29 (24.17%)
**Body Map (CWP(M))**	**N**	363	242	121

	**# CWP(M) positive (%)**	16 (4.41)	11 (4.55%)	5 (4.13%)
	**# CWP(M) negative (%)**	347 (95.59)	231 (95.45%)	116 (95.87%)
**Body Map**	**Zero painful regions reported**	101 (27.82)	62 (25.62%)	39 (32.23%)
	**Reported pain in replaced knee**	194 (53.44)	128 (52.89%)	66 (54.55%)
	**Reported pain in ONLY replaced knee (no other regions selected)**	23 (6.34)	14 (5.79%)	9 (7.44%)
	**Reported pain in one region (excl. replaced knee)**	68 (18.73)	49 (20.25%)	19 (15.70%)
	**Reported pain in two regions (excl. replaced knee)**	36 (9.92)	24 (9.92%)	12 (9.92%)
	**Reported pain in three regions (excl. replaced knee)**	48 (13.22)	34 (14.05%)	14 (11.57%)
	**Reported pain in four regions (excl. replaced knee)**	20 (5.51)	15 (6.20%)	5 (4.13%)
	**Reported pain in five or more region (excl. replaced knee)**	67 (18.46)	44 (18.18%)	23 (19.01%)
**Outcome Measures**				

	N	Mean (SD)	N	Mean (SD)	N	Mean (SD)
**BPI Severity**	363	5.24 (1.69)	242	5.28 (1.71)	121	5.15 (1.65)
**BPI Interference**	363	6.28 (1.91)	242	6.27 (1.91)	121	6.27 (1.93)
**OKS**	363	18.23 (5.83)	242	18.04 (6.05)	121	18.61 (5.36)
**OKS – Pain standardised subscale**	363	36.75 (12.70)	242	36.35 (12.97)	121	37.53 (12.17)
**OKS – Function standardised subscale**	363	39.70 (14.28)	242	39.29 (14.63)	121	40.50 (13.59)
**Pain Catastrophizing scale; N, median [IQR] due to distribution of data**	360	18 [9.25, 30.50]	240	19 [10,31]	120	17 [8, 29]
**PCS: Rumination**	360	7.92 (4.80)	240	8.25 (4.81)	120	7.26 (4.72)
**PCS: Magnification**	360	3.29 (3.20)	240	3.43 (3.26)	120	3.04 (3.07)
**PCS: Helplessness**	360	9.10 (6.17)	240	9.25 (6.34)	120	8.82 (5.84)
**PaSol: Solving Pain**	362	17.15 (5.83)	242	17.25 (5.58)	120	16.94 (6.33)
**PaSol: Meaningful life**	362	21.71 (5.97)	242	21.70 (5.86)	120	21.72 (6.20)
**PaSol: Acceptance of pain**	358	7.89 (4.73)	241	8.00 (4.69)	117	7.65 (4.84)
**PaSol: Belief in solution**	359	8.57 (3.21)	240	8.59 (3.16)	119	8.55 (3.05)
**Patient Satisfaction**	360	62.88 (18.99)	242	63.01 (19.61)	118	62.61 (17.72)
**ICECAP-A**	362	0.72 (0.20)	241	0.72 (0.2)	121	0.74 (0.21)
**Short form-12 (physical)**	363	33.44 (6.51)	242	32.67 (6.50)	121	34.38 (6.44)
**Short form-12 (mental)**	363	42.19 (11.12)	242	42.18 (11.16)	121	42.19 (11.08)
**DN-4 raw score**	359	3.79 (1.71)	240	3.81 (1.70)	119	3.74 (1.75)
**PainDETECT raw score**	363	18.19 (6.77)	242	18.51 (6.69)	121	17.55 (6.90)

Table 3: Demographics and patients who did and did not complete screening

	Screening at 10 weeks<br>N = 5036		Screening at 12 weeks<br>N = 907	
	**Responders**	**Non-responders**	**Responders**	**Non-responders**
N (%)	3058 (61%)	1977 (39%)	553 (61%)	354 (39%)
Mean age (SD)	69.7 (8.8)	69.9 (9.8)	67.7 (8.6)	69.4 (10.4)
% females	54.5%	62.2%	56.2%	62.0%

Table 4: Baseline questionnaire completion by group

	# questionnaires completed sufficiently to produce outcome measure			
	**Intervention**		**Control**	
**Number of expected questionnaires**	**242**		**121**	
	**N**	**%**	**N**	**%**
BPI Severity	242	100%	121	100%
BPI Interference	242	100%	121	100%
OKS	242	100%	121	100%
PainDETECT	242	100%	121	100%
DN-4	240	99%	119	98%
Patient Satisfaction	242	100%	118	98%
Short form-12 physical	242	100%	121	100%
Short form-12 mental	242	100%	121	100%
HADS anxiety	242	100%	121	100%
HADS depression	242	100%	121	100%
ICECAP-A	241	100%	121	100%
Pain Catastrophizing scale	240	99%	121	100%
PaSol solve	242	100%	120	99%
PaSol meaning	242	100%	120	99%
PaSol accept	241	100%	117	97%
PaSol belief	240	99%	119	98%
Body Map (CWP(M))	242	100%	121	100%

Table 5: 6 month questionnaire completion by group

	# questionnaires completed sufficiently to produce outcome measure			
	**Intervention**		**Control**	
**Number of expected questionnaires**	**232**		**116**	
	**N**	**%**	**N**	**%**
BPI Severity	213	92%	101	87%
BPI Interference	213	92%	102	88%
OKS	209	90%	99	85%
PainDETECT	209	90%	100	86%
DN-4	202	87%	96	83%
Patient Satisfaction	206	89%	96	83%
Short form-12 physical	209	90%	100	86%
Short form-12 mental	209	90%	98	84%
HADS anxiety	206	89%	99	85%

HADS depression	206	89%	98	84%
ICECAP-A	206	89%	99	85%
Pain Catastrophizing scale	201	87%	96	83%
PaSol solve	204	88%	95	82%
PaSol meaning	204	88%	96	83%
PaSol accept	203	88%	93	80%
PaSol belief	202	87%	96	83%
Body Map (CWP(M))	215	93%	104	90%

Table 6: 12 month questionnaire completion by group

	**# questionnaires completed sufficiently to produce outcome measure**			
	**Intervention**		**Control**	
**Number of expected questionnaires**	**226**		**111**	
	**N**	**%**	**N**	**%**
BPI Severity	212	94%	100	90%
BPI Interference	213	94%	100	90%
OKS	201	89%	93	84%
PainDETECT	198	88%	94	85%
DN-4	195	86%	94	85%
Patient Satisfaction	197	87%	89	80%
Short form-12 physical	195	86%	93	84%
Short form-12 mental	195	86%	93	84%
HADS anxiety	198	88%	92	83%
HADS depression	197	87%	90	81%
ICECAP-A	197	87%	91	82%
Pain Catastrophizing scale	195	86%	91	82%
PaSol solve	193	85%	89	80%
PaSol meaning	193	85%	90	81%
PaSol accept	191	85%	87	78%
PaSol belief	193	85%	90	81%
Body Map (CWP(M))	213	94%	100	90%

Table 7: Withdrawal summary by group

	**# patients randomised**	**# withdrawals before or at the 6 months follow-up**	**# withdrawals after 6 month follow-up**	**Total withdrawals post-randomisation**
**Intervention**	**242**	**9**	**6**	**15 (6%)**
**Control**	**121**	**5**	**3**	**8 (7%)**
**Overall**	**363**	**14**	**9**	**23 (6%)**

Table 8: Reasons for withdrawal

	**# withdrawals**	**Reason for withdrawal**
**Intervention**	**9**	Patient did not want to be part of the trial
	**2**	New health problems
	**1**	Patient became uncontactable
	**1**	Involved in a conflicting trial
	1	Surgeon advised to withdraw
	1	Incorrect screening – patient did not have TKR
**Control**	4	Patient did not want to be part of the trial
	2	Unhappy with care
	1	Patient became uncontactable
	1	Patient did not think they were part of the trial

Table 9: Number of follow up calls received

Number of calls (X)	Number of patients to receive maximum of X number of calls
0	17 (7%)
1	37 (15%)
2	35 (14%)
3	67 (28%)
4	30 (12%)
5	27 (11%)
6	29 (12%)

Table 10: Timings of follow up calls received

	Weeks to call from recruitment median [IQR]
1st call	11 [9, 14]
2nd call	18 [16, 23]
3rd call	28 [23, 35]
4th call	31 [28, 39]
5th call	37 [34, 43]
6th call	43 [39, 48]

Table 11: Protocol deviations

**Protocol deviation detail (post randomisation)**	**Site**	**Group**	**No participants affected**
Follow-up calls not completed as per protocol	Site5	Intervention	23
Outside window for intervention delivery	Site8	Intervention	10
Non-referral, HADS indications, reasons not documented	Site4	Intervention	5
Non-referral, neuropathic pain indication, reasons not documented	Site4	Intervention	4
Participant declined to attend intervention	Site8	Intervention	4
Outside window for intervention delivery	Site3	Intervention	2
Outside window for intervention delivery	Site4	Intervention	2
Data collected after trial closure	Site5	Intervention	1
Met exclusion criteria - withdrawn	Site5	Intervention	1
Operating surgeon made unannounced contribution to assessment	Site2	Intervention	1
Outside window for intervention delivery	Site1	Intervention	1
Participant declined to attend intervention	Site5	Intervention	1
Referral to GP for anxiety and depression not discussed with participant	Site2	Intervention	1

Table 12: Summary statistics at 6 months and 12 months

	**6 months**		**12 months**	
**N**	**332**		**313**	
	**N**	**Mean (SD)**	**N**	**Mean (SD)**
**BPI Severity**	314	3.71 (2.37)	312	3.31 (2.47)
**BPI Interference**	315	4.15 (2.66)	313	3.70 (2.83)
**OKS**	308	25.72 (9.28)	294	28.03 (10.07)
**OKS – Pain standardised subscale**	308	54.06 (20.57)	294	59.71 (22.52)
**OKS – Function standardised subscale**	309	52.86 (19.85)	295	56.45 (20.99)
**Pain Catastrophizing scale**	297	9 (3, 22)	286	8 (1, 21)

**PCS: Rumination**		298	5.03 (4.90)	287	4.56 (4.78)
**PCS: Magnification**		298	2.38 (2.72)	286	2.33 (2.79)
**PCS: Helplessness**		297	6.12 (6.02)	287	5.66 (6.20)
**PaSol: Solving Pain**		299	13.84 (7.61)	282	12.85 (8.04)
**PaSol: Meaningful life**		300	20.19 (7.95)	283	19.39 (8.71)
**PaSol: Acceptance of pain**		296	8.84 (5.34)	278	8.55 (5.68)
**PaSol: Belief in solution**		298	6.73 (3.87)	283	5.80 (4.34)
**Patient Satisfaction**		302	68.54 (21.85)	286	70.36 (22.92)
**ICECAP-A**		305	0.77 (0.20)	288	0.78 (0.20)
**Short form-12 (physical)**		309	36.44 (8.11)	288	37.46 (9.78)
**Short form-12 (mental)**		307	47.04 (11.53)	288	48.23 (11.09)
**EQ-5D-5L**		313	0.54 (0.24)	310	0.56 (0.25)
**DN-4 raw score**		298	3.38 (1.89)	289	3.03 (2.09)
**PainDETECT raw score**		309	13.97 (6.97)	292	12.81 (7.63)
**DN-4 (Neuropathic pain?)**	**N**	298		289	
	**Yes; N (%)**	192 (64.43%)		164 (56.75%)	
	**No; N (%)**	106 (35.57%)		125 (43.25%)	
**PainDETECT (Neuropathic pain?)**	**N**	309		292	
	**Unlikely; N (%)**	131 (42.39%)		157 (53.77%)	
	**Ambiguous; N (%)**	90 (29.13%)		62 (21.23%)	
	**Likely; N (%)**	88 (28.48%)		73 (25.00%)	
**HADS: Anxiety**	**N**	305		290	
	**Number of “Normal” (%)**	198 (64.92%)		184 (63.45%)	
	**# “Borderline” (%)**	43 (14.10%)		53 (18.28%)	
	**# “Clinical” (%)**	64 (20.98%)		53 (18.28%)	
**HADS: Depression**	**N**	304		287	
	**Number of “Normal” (%)**	183 (60.20%)		182 (63.41%)	
	**# “Borderline” (%)**	71 (23.36%)		70 (24.39%)	
	**# “Clinical” (%)**	50 (16.45%)		35 (12.20%)	
**Frequency of pain Q1[2]**	**N**	315		313	
	**# “Rarely” (%)**	49 (15.56%)		76 (24.28%)	
	**# “Sometimes” (%)**	88 (27.94%)		91 (29.07%)	
	**# “Often” (%)**	72 (22.86%)		61 (19.49%)	
	**# “Most of the time” (%)**	72 (22.86%)		53 (16.93%)	
	**# “All of the time” (%)**	34 (10.79%)		32 (10.22%)	
**Frequency of pain Q2[3]**	**N**	309		291	
	**# “Rarely” (%)**	30 (9.71%)		61 (20.96%)	
	**# “Sometimes” (%)**	98 (31.72%)		76 (26.12%)	
	**# “Often” (%)**	64 (20.71%)		65 (22.34%)	
	**# “Most of the time” (%)**	83 (26.86%)		62 (21.31%)	
	**# “All of the time” (%)**	34 (11.00%)		27 (9.28%)	
**How satisfied are you with the results of your surgery**	**N**	304		289	
	**Very dissatisfied n (%)**	96 (31.58%)		27 (9.34%)	
	**Somewhat dissatisfied n (%)**	124 (40.79%)		52 (17.99%)	

	Somewhat satisfied n (%)	63 (20.72%)	65 (32.87%)
	Very satisfied n (%)	21 (6.91%)	115 (39.79%)
How satisfied are you with the results of your surgery for improving your pain	N	304	288
	Very dissatisfied n (%)	98 (32.24%)	36 (12.50%)
	Somewhat dissatisfied n (%)	101 (33.22%)	48 (16.67%)
	Somewhat satisfied n (%)	74 (24.34%)	87 (30.21%)
	Very satisfied n (%)	31 (10.20%)	117 (40.63%)
How satisfied are you with the results of your surgery for improving your ability to do housework or gardening	N	302	286
	Very dissatisfied n (%)	65 (21.52%)	42 (14.69%)
	Somewhat dissatisfied n (%)	93 (30.79%)	82 (28.67%)
	Somewhat satisfied n (%)	98 (32.45%)	99 (34.62%)
	Very satisfied n (%)	46 (15.23%)	63 (22.03%)
How satisfied are you with the results of your surgery for improving your ability to do leisure activities	N	301	285
	Very dissatisfied n (%)	60 (19.93%)	49 (17.19%)
	Somewhat dissatisfied n (%)	89 (29.57%)	78 (27.37%)
	Somewhat satisfied n (%)	103 (34.22%)	99 (34.74%)
	Very satisfied n (%)	49 (16.28%)	59 (20.70%)
Comparison of pain[4]	N	304	289
	# “Much Better” (%)	141 (46.38%)	155 (53.63%)
	# “A bit better” (%)	63 (20.72%)	60 (20.76%)
	# “The same” (%)	41 (13.49%)	26 (9.00%)
	# “A bit worse” (%)	27 (8.88%)	27 (9.34%)
	# “Much worse” (%)	32 (10.53%)	21 (7.27%)313
Body Map (CWP(M))	N	332	313
	# CWP(M) positive (%)	12 (3.61%)	16 (5.11%)
	# CWP(M) negative (%)	320 (96.97%)	297 (94.89%)
Body Map	Zero painful regions reported	110 (33.13%)	105 (33.55%)
	Reported pain in replaced knee	128 (38.55%)	126 (40.26%)
	Reported pain in ONLY replaced knee (no other regions selected)	17 (5.12%)	17 (5.43%)
	Reported pain in one region (excl. replaced knee)	51 (15.36%)	46 (14.70%)
	Reported pain in two regions (excl. replaced knee)	41 (12.35%)	31 (9.90%)
	Reported pain in three regions (excl. replaced knee)	33 (9.94%)	31 (9.90%)
	Reported pain in four regions (excl. replaced knee)	12 (3.61%)	14 (4.47%)
	Reported pain in five or more region (excl. replaced knee)	68 (20.48%)	69 (22.04%)

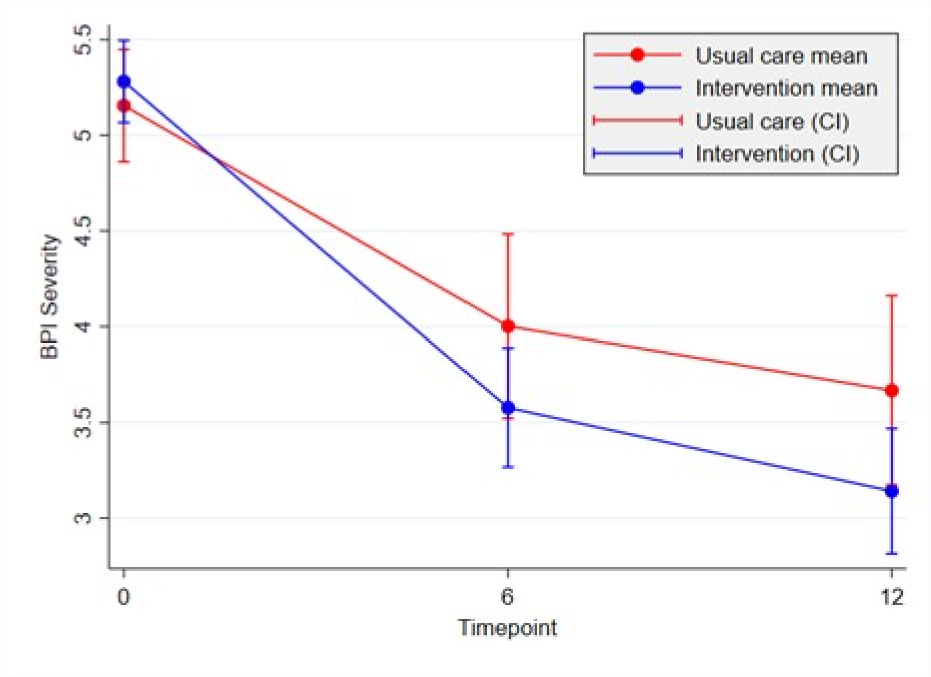

Figure 1: Unadjusted mean BPI severity score with confidence intervals at baseline and follow-up

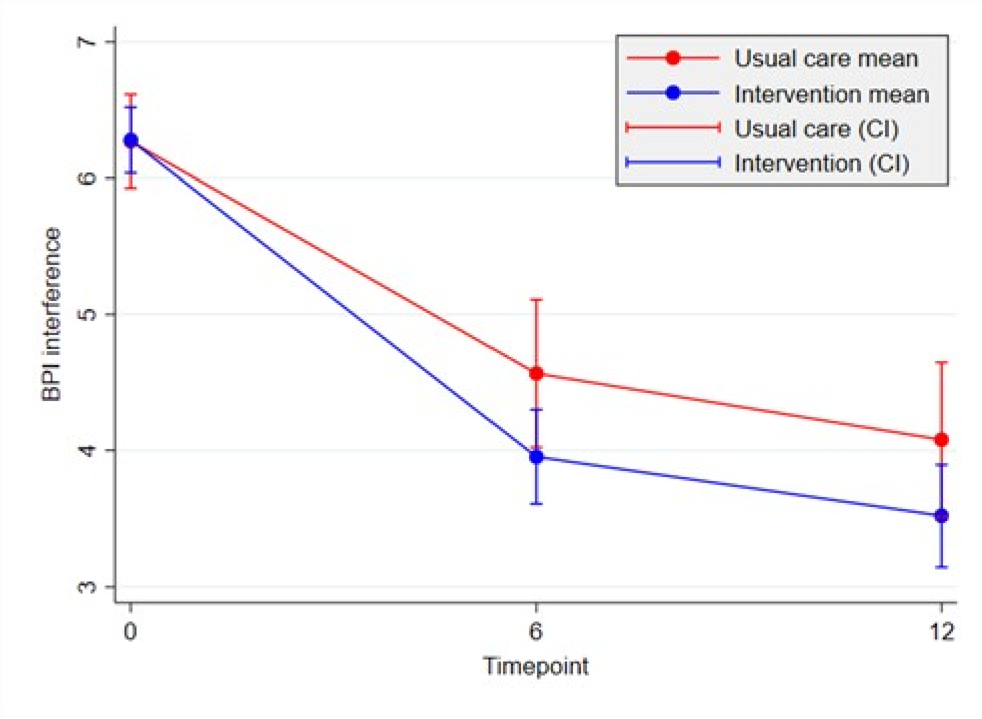

Figure 2: Unadjusted mean BPI interference score with confidence intervals at baseline and follow-up

Table 13: Secondary analysis – adjusting for 'time to follow up from recruitment"

	**Difference in means**[1]	**95% CI**	**P-value**
**BPI Severity**	-0.65	(-1.17, -0.13)	0.015
**BPI Interference**	-0.68	(-1.28, -0.08)	0.028

[1] Adjusted for trial centre and baseline BPI subscores and 'time to follow up'

Sensitivity analysis for primary endpoint

Comparison of results of 'as randomised' analysis of complete cases with 'as randomised' analysis where missing data were imputed using "best" and "worst" case scenarios and the method of mice for primary outcome of **BPI Severity Score**.

Table 14:Sensitivity analysis for missing data

	N	Difference in means[a]	95% CI	p-value
**Complete case**	312	-0.65	(-1.17, -0.13)	0.014
**"Best" case scenario**	363	-0.36	(-0.88, 0.17)	0.186
**"Worst" case scenario**	363	-0.85	(-1.51, -0.20)	0.011
**mice**	363	-0.60	(-1.14, -0.06)	0.030

[a] *Adjusted for trial centre and for baseline BPI subscores*

Comparison of results of 'as randomised' analysis of complete cases with 'as randomised' analysis where missing data were imputed using "best" and "worst" case scenarios and the method of mice for primary outcome of **BPI Interference Score**.

Table 15: Sensitivity analysis for missing data

	N	Difference in means[a]	95% CI	p-value
**Complete case**	313	-0.68	(-1.28, -0.08)	0.026
**"Best" case scenario**	363	-0.33	(-0.94, 0.27)	0.278
**"Worst" case scenario**	363	-0.87	(-1.55, -0.19)	0.013
**mice**	363	-0.57	(-1.19, 0.05)	0.073

[a] *Adjusted for trial centre and for baseline BPI subscores*

Sensitivity analysis – Overlap of patient sample with other interventional trials

Comparison of results of 'as randomised' analysis of all cases with 'as randomised' analysis where only patients involved in STAR are analysed for primary outcome of BPI Severity scale.

Table 16: Overlap sensitivity analysis for BPI Severity scale

	N	Difference in means[a]	95% CI	p-value
Overall 'as randomised' analysis	312	-0.65	(-1.17, -0.13)	0.014
Excluding patients in similar interventional trials	308	-0.65	(-1.17, -0.14)	0.014

[a] *Adjusted for trial centre and for baseline BPI subscores*

Per protocol and CACE analysis – primary outcomes

- 9 intervention patients did not have an assessment clinic. 9/363 = 97.52% and so CACE is not necessary according to section 6.4 Compliance of the SAP.

Comparison of results of 'as randomised' analysis of complete cases with per protocol analysis and CACE analysis for primary outcome of **BPI Severity Score**.

Table 17: Sensitivity analysis for missing data

	N	Difference in means[a]	95% CI	p-value
'as randomised'	312	-0.65	(-1.17, -0.13)	0.014
**Per protocol**	308	-0.67	(-1.19, -0.15)	0.011

[a] *Adjusted for trial centre and for baseline BPI subscores*

Comparison of results of 'as randomised' analysis of complete cases with per protocol analysis and CACE for primary outcome of **BPI Interference Score**.

Table 18: Sensitivity analysis for missing data

	N	Difference in means[a]	95% CI	p-value
'as randomised'	313	-0.68	(-1.28, -0.08)	0.026
**Per protocol**	309	-0.71	(-1.31, -0.10)	0.022

[a] *Adjusted for trial centre and for baseline BPI subscores*

__Sensitivity analysis – time between TKR operation and assessment clinic__

- The median (IQR) number of months between TKR operation and assessment clinic is 3.61 (3.32, 4.01). 19 (8%) intervention patients had their assessment clinic withing 3 months of the TKR operation. 172 (72%) intervention patients had their assessment clinic withing 4 months of the TKR operation.

Table 19: Sensitivity analysis excluding patients who had their assessment after 4 months post-operative

	Excluding those patients who had their assessment after 4 months post-operative			
	N	Difference in means	95% CI	P-value
**BPI Pain subscale**	255	-0.67	(-1.23, -0.11)	0.019
**BPI Interference subscale**	256	-0.76	(-1.41, -0.11)	0.023

__Repeated measures analysis__

Table 20: Repeated measures analysis of BPI subscales

	6 months			12 months			
	Difference in means	95% CI	P-value	Difference in means	95% CI	P-value	P-value of interaction
**BPI Pain subscale**	-0.55	(-1.05, -0.06)	0.028	-0.62	(-1.11, -0.13)	0.014	0.746
**BPI Interference subscale**	-0.71	(-1.28, -0.15)	0.014	-0.61	(-1.18, -0.04)	0.034	0.708

**adjusted for: trial centre and baseline BPI subscores*

__Subgroup analyses__

Table 21: Evidence of interaction

Subgroup variable	Outcome	p-value of interaction
Trial Centre	BPI severity	0.287
	BPI Interference	0.154
OKS continuous	BPI severity	0.022
	BPI Interference	0.002
OKS categorical	BPI severity	0.365
	BPI Interference	0.521
PaSol composite continuous	BPI severity	0.680
	BPI Interference	0.647
PaSol composite categorical	BPI severity	0.533
	BPI Interference	0.234

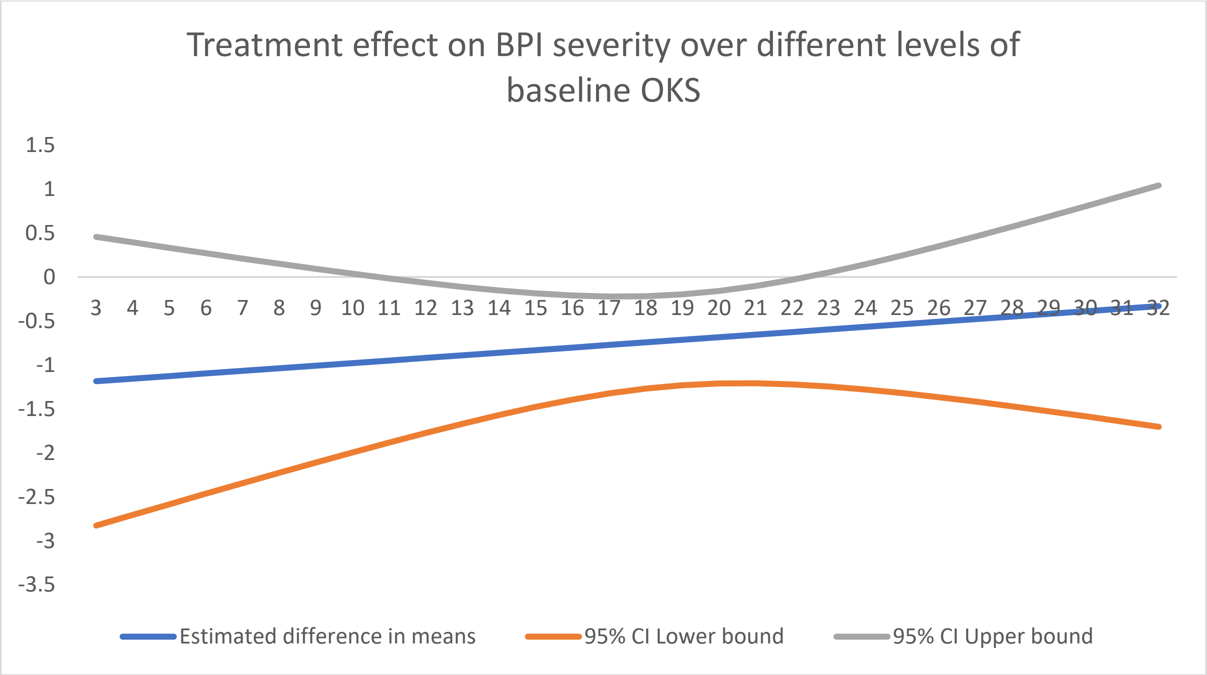

Figure 3: Treatment effect on BPI severity over differing levels of OKS (continuous) at baseline

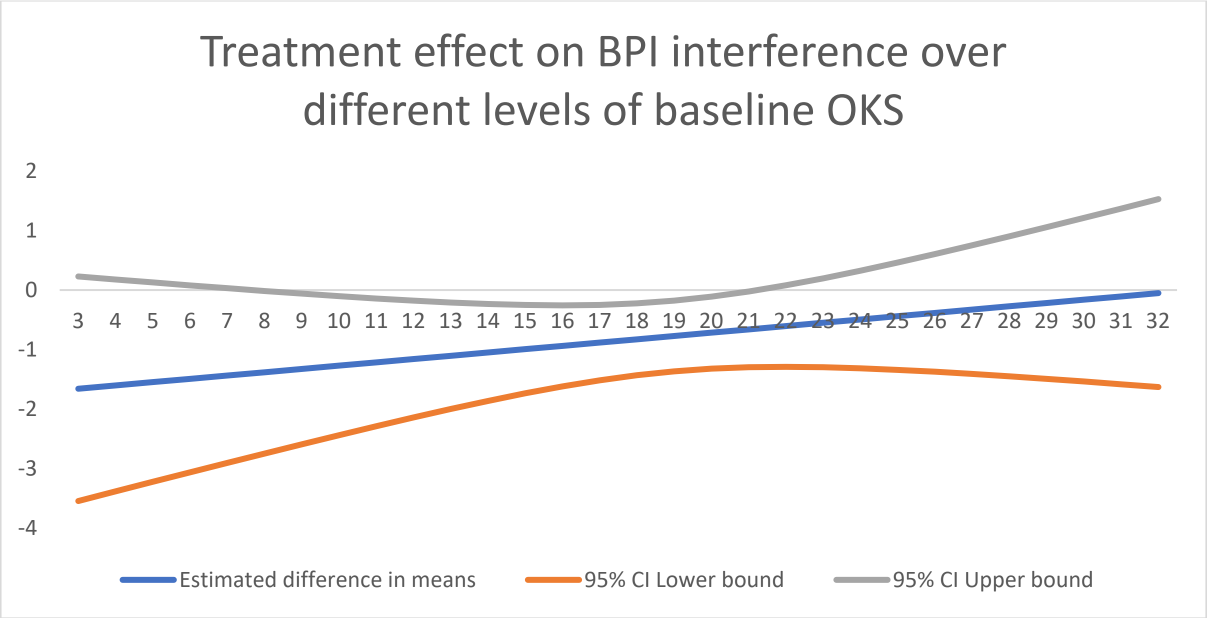

Figure 4: Treatment effect on BPI interference over differing levels of OKS (continuous) at baseline

Table 22: Subgroup analysis of OKS categories

Outcome	OKS category	N	Difference in means	95% CI
**BPI Pain subscale**	Severe	171	-1.00	(-1.70, -0.29)
	Moderate	134	-0.19	(-0.97, 0.60)
	Mild - moderate	7	-1.49	(-5.09, 2.11)
**BPI Interference subscale**	Severe	172	-1.09	(-1.91, -0.27)
	Moderate	134	-0.18	(-1.09, 0.73)
	Mild - moderate	7	-0.53	(-4.71, 3.66)

Accounting for potential impact of COVID-19

Table 23: Accounting for potential impact of COVID-19: Psychological outcomes

	Baseline				12 months				Difference (12 month outcome – baseline outcome)			
	12 month completion before the 23/03/2020		12 month completion on or after the 23/03/2020		12 month completion before the 23/03/2020		12 month completion on or after the 23/03/2020		12 month completion before the 23/03/2020		12 month completion on or after the 23/03/2020	
	N	Median (IQR)	N	Median (IQR)	N	Median (IQR)	N	Median (IQR)	N	Median (IQR)	N	Median (IQR)
**HADS anxiety**	274	7 (4, 11)	39	7 (3, 10)	254	6 (2, 9)	36	6 (2, 10)	254	-1 (-3, 1)	36	-1 (-2, 1)
**HADS depression**	274	7.5 (5, 10)	39	6 (3, 10)	252	5.5 (3, 9)	35	6 (2, 8)	252	-2 (-3.5, 1)	35	-2 (-3, 0)
**PaSol Solve**	274	18 (13, 22)	39	18 (12, 20)	246	15 (6, 24)	36	13.5 (1, 20)	246	-3 (-9, 1)	36	-3 (-12, 3)
**PaSol meaning**	274	23 (18, 27)	39	22 (17, 24)	247	21 (15, 27)	36	18 (13.5, 22)	247	0 (-7, 3)	36	-1 (-8.5, 2.5)
**PaSol accept**	272	7 (4, 11)	39	8 (5,12)	242	9 (5, 13)	36	7 (1, 13)	240	1 (-3, 5)	36	-0.5 (-3.5, 2.5)
**PaSol Belief**	271	9 (6, 12)	39	8 (6, 11)	247	6 (1, 10)	36	4 (0, 8.5)	245	-2 (-6, 0)	36	-3 (-6, 0.5)

Secondary outcomes

Table 24: OKS sensitivity analysis: missing data

	**N**	**Difference in means[a]**	**95% CI**	**p-value**
**Complete case**	294	2.68	(0.58, 4.78)	0.013
**"Best" case scenario**	363	0.21	(-2.33, 2.75)	0.871
**"Worst" case scenario**	363	3.36	(0.56, 6.16)	0.019
**mice**	363	2.16	(0.07, 4.24)	0.042

[a] *Adjusted for trial centre and for baseline BPI subscores and baseline OKS*

Table 25: OKS sensitivity analysis: per protocol

	N	Difference in means[a]	95% CI	p-value
'as randomised'	294	2.68	(0.58, 4.78)	0.013
**Per protocol**	290	2.72	(0.60, 4.84)	0.012

[a] Adjusted for trial centre and for baseline BPI subscores

Table 26: DN-4 sensitivity analysis: missing data

	N	Difference in means[a]	95% CI	p-value
**Complete case**	286	-0.10	(-0.55, 0.35)	0.653
**"Best" case scenario**	359	0.12	(-1.34, 0.59)	0.606
**"Worst" case scenario**	359	-0.13	(-0.62, 0.36)	0.607
**mice**	359	-0.05	(-0.52, 0.42)	0.840

[a] Adjusted for trial centre and for baseline BPI subscores and baseline DN-4

Table 27: DN-4 sensitivity analysis: per protocol

	N	Difference in means[a]	95% CI	p-value
'as randomised'	286	-0.10	(-0.55, 0.35)	0.653
**Per protocol**	282	-0.11	(-0.56, 0.35)	0.644

[a] Adjusted for trial centre and for baseline BPI subscores

Table 28: PainDETECT sensitivity analysis: missing data

	N	Difference in means[a]	95% CI	p-value
**Complete case**	292	-0.93	(-2.51, 0.65)	0.249
**"Best" case scenario**	363	0.30	(-1.55, 2.14)	0.753
**"Worst" case scenario**	363	-1.49	(-3.92, 0.94)	0.228
**mice**	363	-0.77	(-2.33, 0.80)	0.335

[a] Adjusted for trial centre and for baseline BPI subscores and baseline PainDETECT

Table 29: PainDETECT sensitivity analysis: per protocol

	N	Difference in means[a]	95% CI	p-value
'as randomised'	292	-0.93	(-2.51, 0.65)	0.249
**Per protocol**	288	-1.03	(-2.62, 0.56)	0.204

[a] Adjusted for trial centre and for baseline BPI subscores

Table 30: PCS sensitivity analysis: missing data

	N	Difference in means[a]	95% CI	p-value
**Complete case**	232	0.90	(0.70, 1.16)	0.428
**"Best" case scenario**	232	0.90	(0.70, 1.16)	0.428
**"Worst" case scenario**	307	0.89	(0.69, 1.14)	0.364
**mice**	360	0.86	(0.67, 1.12)	0.261

[a] Adjusted for trial centre and for baseline BPI subscores and baseline PCS. Analysed on the log scale and back transformed

Table 31: PCS sensitivity analysis: per protocol

	N	Difference in means[a]	95% CI	p-value
'as randomised'	232	0.90	(0.70, 1.16)	0.428
**Per protocol**	228	0.89	(0.69, 1.15)	0.366

[a] Adjusted for trial centre and for baseline BPI subscores . Analysed on the log scale and back transformed

Table 32: PaSol sensitivity analysis: missing data

		N	Difference in means[a]	95% CI	p-value
**Solve**	**Complete case**	282	-1.18	(-3.09, 0.74)	0.226
	**"Best" case scenario**	362	-1.20	(-2.95, 0.55)	0.177
	**"Worst" case scenario**	362	0.19	(-1.74, 2.12)	0.845
	**mice**	362	-0.56	(-1.45, 1.34)	0.564
**Meaning**	**Complete case**	283	-0.64	(-2.83, 1.55)	0.565
	**"Best" case scenario**	362	-0.94	(-2.87, 0.99)	0.338
	**"Worst" case scenario**	362	0.52	(-1.89, 2.94)	0.670
	**mice**	362	-0.30	(-2.45, 1.85)	0.784
**Acceptance**	**Complete case**	276	-0.22	(-1.65, 1.20)	0.757
	**"Best" case scenario**	358	-0.63	(-2.00, 0.73)	0.363
	**"Worst" case scenario**	358	0.38	(-0.99, 1.75)	0.586
	**mice**	358	-0.04	(-1.41, 1.33)	0.959
**Belief**	**Complete case**	281	-0.38	(-1.47, 0.70)	0.490
	**"Best" case scenario**	359	-0.54	(-1.54, 0.46)	0.285
	**"Worst" case scenario**	359	0.10	(-0.89, 1.09)	0.839
	**mice**	359	-0.26	(-1.36, 0.85)	0.645

[a] *Adjusted for trial centre and for baseline BPI subscores and respective baseline PaSol subscore*

Table 33: PaSol sensitivity analysis: per protocol

		N	Difference in means[a]	95% CI	p-value
**Solving Pain**	'as randomised'	282	-1.18	(-3.09, 0.74)	0.226
	**Per protocol**	278	-1.36	(-3.28, 0.56)	0.163
**Meaningful life**	'as randomised'	283	-0.64	(-2.83, 1.55)	0.565
	**Per protocol**	279	-0.76	(-2.96, 1.45)	0.500
**Acceptance of pain**	'as randomised'	276	-0.22	(-1.65, 1.20)	0.757
	**Per protocol**	272	-0.37	(-1.81, 1.06)	0.608
**Belief in solution**	'as randomised'	281	-0.38	(-1.47, 0.70)	0.490
	**Per protocol**	277	-0.44	(-1.54, 0.65)	0.426

[a] *Adjusted for trial centre and for baseline BPI subscores*

Table 34: Patient satisfaction sensitivity analysis: missing data

	N	Difference in means[a]	95% CI	p-value
**Complete case**	284	3.79	(-1.47, 9.06)	0.157
**"Best" case scenario**	360	-0.32	(-5.39, 4.76)	0.902
**"Worst" case scenario**	360	5.55	(-0.08, 11.18)	0.053
**mice**	360	3.61	(-1.90, 9.11)	0.197

[a] *Adjusted for trial centre and for baseline BPI subscores and baseline Satisfaction score*

Table 35: Patient satisfaction sensitivity analysis: per protocol

	N	Difference in means[a]	95% CI	p-value
'as randomised'	284	3.79	(-1.47, 9.06)	0.157
**Per protocol**	280	4.17	(-1.12, 9.45)	0.122

[a] *Adjusted for trial centre and for baseline BPI subscores*

Table 36: ICECAP-A sensitivity analysis: missing data

	N	Difference in means[a]	95% CI	p-value
**Complete case**	287	0.03	(-0.004, 0.06)	0.085
**"Best" case scenario**	362	-0.004	(-0.04, 0.03)	0.839
**"Worst" case scenario**	362	0.06	(-0.005, 0.13)	0.070
**mice**	362	0.02	(-0.02, 0.05)	0.381

[a] *Adjusted for trial centre and for baseline BPI subscores and baseline ICECAP-A*

Table 37: ICECAP-A sensitivity analysis: per protocol

	N	Difference in means[a]	95% CI	p-value
'as randomised'	287	0.03	(-0.004, 0.06)	0.085
**Per protocol**	283	0.03	(-0.004, 0.06)	0.080

[a] *Adjusted for trial centre and for baseline BPI subscores*

Table 38: SF-12 sensitivity analysis: missing data

		N	Difference in means[a]	95% CI	p-value
**Physical**	**Complete case**	288	2.07	(-0.10, 4.23)	0.061
	**"Best" case scenario**	363	-1.39	(-7.28, 4.50)	0.643
	**"Worst" case scenario**	363	3.12	(-0.51, 6.76)	0.092
	**mice**	363	1.49	(-0.67, 3.66)	0.176
**Mental**	**Complete case**	288	-0.08	(-2.29, 2.12)	0.940
	**"Best" case scenario**	363	-2.22	(-7.27, 2.83)	0.388
	**"Worst" case scenario**	363	1.58	(-2.86, 6.02)	0.485
	**mice**	363	-0.17	(-2.35, 2.01)	0.878

[a] *Adjusted for trial centre and for baseline BPI subscores and respective baseline SF-12 subscore*

Table 39: SF-12 sensitivity analysis: per protocol

		N	Difference in means[a]	95% CI	p-value
**Short form-12 (physical)**	'as randomised'	288	2.07	(-0.10, 4.23)	0.061
	**Per protocol**	284	2.05	(-0.13, 4.22)	0.066
**Short form-12 (mental)**	'as randomised'	288	-0.08	(-2.29, 2.12)	0.940
	**Per protocol**	284	0.09	(-2.11, 2.29)	0.933

[a] *Adjusted for trial centre and for baseline BPI subscores*

Table 40: HADS anxiety scale sensitivity analysis: missing data

	N	Difference in means[a]	95% CI	p-value
**Complete case**	290	-0.69	(-1.47, 0.08)	0.079
**"Best" case scenario**	363	0.16	(-0.77, 1.08)	0.741
**"Worst" case scenario**	363	-1.26	(-2.63, 0.11)	0.071
**mice**	363	-0.56	(-1.33, 0.21)	0.150

[a] *Adjusted for trial centre and for baseline BPI subscores and HADS anxiety*

Table 41: HADS anxiety scale sensitivity analysis: per protocol

	N	Difference in means[a]	95% CI	p-value
'as randomised'	290	-0.70	(-1.47, 0.08)	0.079
**Per protocol**	286	-0.73	(-1.51, 0.04)	0.064

[a] *Adjusted for trial centre and for baseline BPI subscores*

Table 42: HADS depression scale sensitivity analysis: missing data

	N	Difference in means[a]	95% CI	p-value
**Complete case**	287	-0.69	(-1.47, 0.10)	0.086
**"Best" case scenario**	363	0.16	(-0.73, 1.05)	0.719
**"Worst" case scenario**	363	-1.50	(-2.89, -0.11)	0.035
**mice**	363	-0.52	(-1.28, 0.23)	0.173

[a] *Adjusted for trial centre and for baseline BPI subscores and baseline HADS depression*

Table 43: HADS depression scale sensitivity analysis: per protocol

	N	Difference in means[a]	95% CI	p-value
'as randomised'	287	-0.69	(-1.47, 0.10)	0.086
**Per protocol**	283	-0.72	(-1.51, 0.08)	0.077

[a] *Adjusted for trial centre and for baseline BPI subscores*

Table 44: Chronic widespread pain sensitivity analysis: missing data

	N	Odds Ratio[a]	95% CI	p-value
**Complete case**	280	0.61[a]	(0.20, 1.91)	0.399
**"Best" case scenario**	325	0.59[b]	(0.20, 1.71)	0.328
**"Worst" case scenario**	363	0.61[a]	(0.34, 1.08)	0.008
**mice**	363	0.65[a]	(0.22, 1.90)	0.432

[a] *Adjusted for trial centre and for baseline BPI subscores and baseline CWP*
[b] *Adjusted for baseline BPI subscores and baseline CWP*

Table 45: Chronic widespread pain sensitivity analysis: per protocol

	N	Odds Ratio[a]	95% CI	p-value
'as randomised'	313	0.61	(0.20, 1.91)	0.399
**Per protocol**	307	0.60	(0.32, 1.14)	0.122

[a] Adjusted for trial centre and for baseline BPI subscores

Table 46: Frequency of pain sensitivity analysis: missing data

		N	Odds Ratio[a]	95% CI	p-value
**Section A: Question 5**	**Complete case**	311	0.64	(0.34, 1.21)	0.170
	**"Best" case scenario**	361	0.95	(0.59, 1.52)	0.836
	**"Worst" case scenario**	361	0.61	(0.33, 1.13)	0.120
	**mice**	361	0.62	(0.34, 1.14)	0.123
**Section D: Question 8**	**Complete case**	291	0.55	(0.27, 1.11)	0.095
	**"Best" case scenario**	362	0.92	(0.57, 1.47)	0.724
	**"Worst" case scenario**	362	0.59	(0.30, 1.16)	0.124
	**mice**	362	0.62	(0.32, 1.20)	0.155

[a] Adjusted for trial centre and for baseline BPI subscores and baseline freq. of pain
[b] Adjusted for baseline BPI subscores and baseline freq. of pain

Table 47: Frequency of pain sensitivity analysis: per protocol

		N	Odds Ratio[a]	95% CI	p-value
**Section A: Question 5**	'as randomised'	311	0.64	(0.34, 1.21)	0.170
	**Per protocol**	307	0.60	(0.32, 1.14)	0.122
**Section D: Question 8**	'as randomised'	291	0.55	(0.27, 1.11)	0.095
	**Per protocol**	287	0.54	(0.27, 1.11)	0.094

[a] Adjusted for trial centre and for baseline BPI subscores

Table 48: Comparison of pain to pre-operative pain sensitivity analysis: missing data

	N	Odds Ratio[a]	95% CI	p-value
**Complete case**	289	0.62	(0.34, 1.12)	0.113
**"Best" case scenario**	362	0.83	(0.50, 1.36)	0.451
**"Worst" case scenario**	362	0.59	(0.35, 0.98)	0.042
**mice**	362	0.59	(0.36, 0.98)	0.041

[a] Adjusted for trial centre and for baseline BPI subscores and baseline comparison of pain
[b] Adjusted for baseline BPI subscores and baseline CWP

Table 49: Comparison of pain to pre-operative pain sensitivity analysis: per protocol

	N	Odds Ratio[a]	95% CI	p-value
'as randomised'	289	0.62	(0.34, 1.12)	0.113
**Per protocol**	285	0.60	(0.33, 1.10)	0.097

[a] Adjusted for trial centre and for baseline BPI subscores

Table 50: Adverse reactions

Relatedness to trial intervention:		Frequency	Site	Details
Severity:	Not serious	1 (100%)	2	Participant phoned the research team in distress following receipt of her intervention referral letter; a referral for anxiety/depression had not been discussed at her appointment
	Serious unexpected	0		
	Serious expected	0		

Table 51: Responder analysis

	BPI pain severity			BPI interference		
	Intervention	Control	Overall	Intervention	Control	Overall
N available data	212	100	312	213	100	313
Got better by 30% or more	132 (62.26%)	54 (54.00%)	186 (59.62%)	135 (63.38%)	59 (59.00%)	194 (61.98%)
Stayed withing a +/- 30% difference	67 (31.60%)	33 (33.00%)	100 (32.05%)	70 (32.86%)	35 (35.00%)	105 (33.55%)
Got worse by 30% or more	13 (6.13%)	13 (13.00%)	26 (8.33%)	8 (3.76%)	6 (6.00%)	14 (4.47%)

Table 52 Studies from systematic review

**Study**<br>**Country, date of recruitment**	**Inclusion**<br>**Number randomised (intervention; control)**	**Intervention**<br>**Comparator**	**Risk of bias issues**<br>**Key results**
Published studies			
Singh et al. 2010 [1]<br>USA, 2006-2009	Pain after total knee replacement for >3 months, NRS pain intensity ≥6/10<br>N=54: 60 knees (30:30)	Single intra-articular botulinum toxin A injection<br>Single intra-articular injection of saline	Low risk of bias<br>Reduced pain intensity in botulinum A group after 3 months. Pain relief to about 40 days
Ma et al. 2016 [2]<br>China, 2014-2015	Intractable pain of knee joint after total knee replacement<br>N=100 (50:50)	Denervation therapy<br>Drug treatment	No losses to follow up<br>Denervation therapy associated with improved symptoms
Pickering et al. [3]<br>France, 2016	Localized neuropathic pain after knee surgery<br>N=36 (24;12)	5% lidocaine-medicated plaster for 3 months<br>5% plaster with no drug for 3 months	No losses to follow up<br>Lidocaine plaster reduced localized neuropathic pain
Qudsi-Sinclair et al. 2017 [4]<br>Spain, 2012-2014	Pain after total knee replacement<br>N=33 (15:18, 14:14 received intervention)	Single radiofrequency genicular nerve block<br>Single analgesic block with corticosteroid	Some concerns: uneven follow up in small study<br>Similar pain outcomes in groups
From trial registries			
NCT02211534 [5]	Persistent post-operative pain following total knee replacement	Pulsed electromagnetic energy field therapy<br>Sham pulsed electromagnetic field	
NCT02931435 [6]	Chronic knee pain despite total knee replacement at least 6 months	Nerve block with radiofrequency ablation<br>Sham radiofrequency ablation	
NCT03825965 [7]	Persistent post-surgical pain following total knee replacement	Cannabinoids<br>Placebo	
NCT04100707 [8]	Knee pain 3 months after total knee replacement	Genicular nerve blocks<br>Sham comparator	
NCT03973177 [9]<br>Crossover	Refractory chronic knee pain for more than 6 months after total knee replacement	Phenol injection: neurolysis of genicular nerves<br>Methylprednisolone injection	
Larsen et al. 2020 [10,11]	Chronic pain after primary total knee replacement	Neuromuscular exercise and pain neuroscience education<br>Pain neuroscience education	

Supplementary Information relating to the economic evaluation

Information received from informatics departments:

Informatics departments provided electronic information for inpatient stays and daycases in the form of: ICD10 (International Statistical Classification of Diseases), OPCS4 (International Classification of Interventions and Procedures), HRG (Health Resource Group) codes, admission and discharge dates; and for outpatient visits, Imaging and Emergency department attendances (if the centre had an A&E department) in the form of: service codes, HRG/Currency codes and attendance dates.

Clinical opinion was used to ensure only admissions, outpatient visits and emergency department attendances which were related to the STAR pathway, pain or knee replacement were included in the analysis. To avoid double counting all outpatient appointments and x rays occurring on the STAR clinic dates were deleted from the informatics data. Each hospital admission was classified into Daycase, short stay (<2 days) and longstay.

Sensitivity analyses:

The following sensitivity analyses were conducted where there was methodological uncertainty, or assumptions were made during the study and analysis:

1) Changing the ratio of direct to indirect time in the calculation of the unit cost for the ESPs.
2) Altering the assumption that the home care worker visit use, would be the same for the whole of the 6 months as it was for one week (as asked in the follow-up questionnaires) to 3 months and 1 month.
3) Costing the Home changes and equipment at a quarter and a half of the initial cost, to take into account that some of the equipment would be returned.
4) Assuming that when it was unclear who paid for the home changes and equipment, the provider was the NHS/PSS.
5) Excluding “other” community-based services such as hydrotherapy, which may have been accounted for in the outpatient attendances.
6) Costing HRG’s using an elective inpatient cost, rather than non-elective inpatient short stay and long stay costs.

Table 53 CHEERS checklist

**Section/item**	**Item**	**Recommendation**	**Reported on page no/line no**
Title and abstract			
Title	1	Identify the study as an economic evaluation or use more specific terms such as “cost-effectiveness analysis”, and describe the interventions compared.	Not applicable as reporting the RCT
Abstract	2	Provide a structured summary of objectives, perspective, setting, methods (including study design and inputs), results (including base case and uncertainty analyses), and conclusions.	Abstract: pg 4-5: note given this is a joint paper,there was not space to include all the methods relating to economic evaluation and the uncertainty analyses
Introduction			
Background andobjectives	3	Provide an explicit statement of the broader context for thestudy.<br><br>Present the study question and its relevance for health policy orpractice decisions.	Background: pg 6-7
Methods			
Target population andsubgroups	4	Describe characteristics of the base case population andsubgroups analysed, including why they were chosen.	Participants:pg7
Setting and location	5	State relevant aspects of the system(s) in which the decision(s) need(s) to be made.	Study design: pg 7
Study perspective	6	Describe the perspective of the study and relate this to the costs being evaluated.	Measurement and valuation of resource use data: pg 12
Comparators	7	Describe the interventions or strategies being compared and state why they were chosen.	Procedures: pg 9-11
Time horizon	8	State the time horizon(s) over which costs and consequences are being evaluated and say why appropriate.	Measurement and valuation of resource use data pg 12. As an RCT based analysis the time horizon was the same for the

			effectiveness and cost-effectiveness measures
Discount rate	9	Report the choice of discount rate(s) used for costs and outcomes and say why appropriate.	N/A
Choice of health outcomes	10	Describe what outcomes were used as the measure(s) of benefit in the evaluation and their relevance for the type ofanalysis performed.	Outcomes: pg12 and reference 26
Measurement of effectiveness	11a	*Single study-based estimates:* Describe fully the design features of the single effectiveness study and why the singlestudy was a sufficient source of clinical effectiveness data.	Methods: pg 7-pg 15. pg 6 line 20
	11b	*Synthesis-based estimates:* Describe fully the methods used foridentification of included studies and synthesis of clinical effectiveness data.	N/A
Measurement and valuation of preference based outcomes	12	If applicable, describe the population and methods used toelicit preferences for outcomes.	N/A
Estimating resources and costs	13a	*Single study-based economic evaluation:* Describe approaches used to estimate resource use associated with the alternative interventions. Describe primary or secondary research methodsfor valuing each resource item in terms of its unit cost.<br><br>Describe any adjustments made to approximate to opportunitycost	Measurement and valuation of resource usedata pg 12. Supplementary material pg 23, Table 55
	13b	*Model-based economic evaluation:* Describe approaches anddata sources used to estimate resource use associated with model health states. Describe primary or secondary research methods for valuing each resource item in terms of its unit cost. Describe any adjustments made to approximate to opportunity costs.	N/A
Currency, price date,and conversion	14	Report the dates of the estimated resource quantities and unitcosts. Describe methods for adjusting estimated unit costs to the year of reported costs if necessary. Describe methods for converting costs into a common currency base and the exchange rate.	Measurement and valuation of resource use data pg 12
Choice of model	15	Describe and give reasons for the specific type of decision-analytical model used. Providing a figure to show model structure is strongly recommended.	N/A
Assumptions	16	Describe all structural or other assumptions underpinning the decision-analytical model.	N/A
Analytical methods	17	Describe all analytical methods supporting the evaluation. This could include methods for dealing with skewed, missing, or censored data; extrapolation methods; methods for pooling data; approaches to validate or make adjustments (such as half cycle corrections) to a model;	Cost-effectiveness analysis: pg 14-15. Supplementary material pg 23

		and methods for handling population heterogeneity and uncertainty.	
Results			
Study parameters	18	Report the values, ranges, references, and, if used, probability distributions for all parameters. Report reasons or sources fordistributions used to represent uncertainty where appropriate. Providing a table to show the input values is strongly recommended.	N/A
Incremental costs andoutcomes	19	For each intervention, report mean values for the main categories of estimated costs and outcomes of interest, as wellas mean differences between the comparator groups. If applicable, report incremental cost-effectiveness ratios.	Results: pg 18 & Table 3
Characterising uncertainty	20a	*Single study-based economic evaluation:* Describe the effectsof sampling uncertainty for the estimated incremental cost andincremental effectiveness parameters, together with the impact of methodological assumptions (such as discount rate, studyperspective).	Results: pg18-19 Supplementary material Table 60
	20b	*Model-based economic evaluation:* Describe the effects on theresults of uncertainty for all input parameters, and uncertainty related to the structure of the model and assumptions.	N/A
Characterising heterogeneity	21	If applicable, report differences in costs, outcomes, or cost- effectiveness that can be explained by variations between subgroups of patients with different baseline characteristics orother observed variability in effects that are not reducible bymore information.	N/A
Discussion			
Study findings, limitations, generalisabiliy, and current knowledge	22	Summarise key study findings and describe how they supportthe conclusions reached. Discuss limitations and the generalisability of the findings and how the findings fit withcurrent knowledge.	Discussion pg19-22
Other			
Source of funding	23	Describe how the study was funded and the role of the funder in the identification, design, conduct, and reporting of the analysis. Describe other non-monetary sources of support.	Role of funding source pg15. Funding pg25

Conflicts of interest	24	Describe any potential for conflict of interest of study contributors in accordance with journal policy. In the absenceof a journal policy, we recommend authors comply with International Committee of Medical Journal Editors recommendations.	Competing Interests: pg24

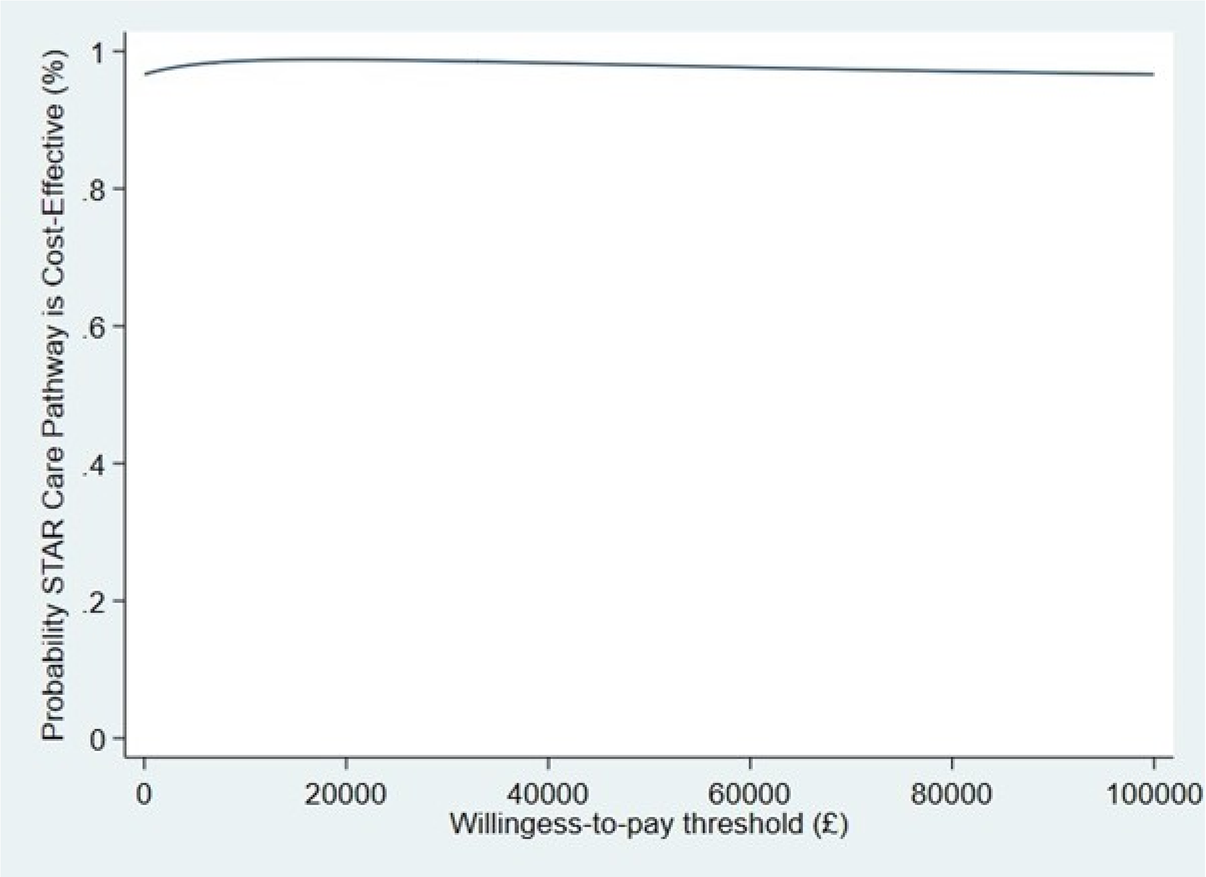

Figure 5: Cost-effectiveness acceptability curve from the NHS/PSS perspective

Table 54 Resources collected and their valuation (2019/20 prices excluding VAT)

**Resource**	**Unit Cost (£)**	**Source of Cost**
STAR pathway assessment clinic (minutes)	Varies[a]	Curtis and Burns (2020)[12]
Inpatient and Daycase admissions	Varies[b,c]	NHS Reference Costs[13]
Outpatient appointments	Varies[c,d]	NHS Reference Costs[13]
Outpatient procedures	Varies[c]	NHS Reference Costs[13]
Radiology investigations	Varies[c]	NHS Reference Costs[13]
Accident and Emergency attendances (no admission)	Varies[c]	NHS Reference Costs[13]
General Practitioner (Surgery)	39.23	Curtis and Burns (2020)[12]
General Practitioner (Home)	124.69[c]	Curtis and Burns (2013)[14]
General Practitioner (Phone)	17.20[e]	Curtis and Burns (2020)[12]
Nurse (Surgery)	10.85[f]	Curtis and Burns (2020)[12]
Nurse (Phone)	4.60[g]	Curtis and Burns (2020)[12]
District Nurse	41.05[c]	Curtis and Burns (2015)[15]
Community physiotherapist	48.00[h]	Curtis and Burns (2020)[12]
Acupuncturist (NHS and private)	Varies[h]	Curtis and Burns (2020)[12] and patient's reported cost
Hydrotherapy session (NHS and private)	Varies[h]	Curtis and Burns (2020)[12] and patient's reported cost
NHS 111 Service	9.19 per call[c]	Turner et al (2012)[16]
Other NHS and Community-based health service contacts	Varies	Curtis and Burns (2020)[12], NHS Reference Costs[13], patient's reported cost
Medications	Varies[i]	British National Formulary[17]
Prescription Charges	9.15 per item	NHS (2020)[18]
Home Care Worker	30.29 per hour[j]	Curtis and Burns (2020)[12]
Home Changes and Equipment	Varies	NRS Healthcare[19], Curtis and Burns (2019)[20] patient reported costs
Wage rate	13.68[k]	ONS Annual Survey of Hours and Earnings (2020)[21]
Community based Travel cost per journey	Varies	The patient's reported mode of transport to each healthcare facility and the fare/miles reported were used to create a mean unit cost for travel to each health facility.
Hospital based Travel cost per journey	Varies	The patient's postcode was used to estimate mileage from the hospital.

[a] Based on the Extended Scope Practitioners pay band, using a ratio of 75% contact to 25% non-contact time. This unit cost includes an allowance for training and study days.
[b] Each admission was assigned an Health Resource Group (HRG) code. The daycase, short stay (<2days) and longstay non elective inpatient reference cost related to the respective HRG code was used.
[c] Costs inflated to 2019-20 values using the NHS cost inflation index (NHSCII)[12]
[d] A unit cost relating to the relevant service code (i.e. speciality) was used.
[e] Based on triage time of 4 minutes.
[f] Based on a 15.5minute consultation[15].
[g] Based on triage time of 6.56 minutes.
[h] NHS costs were based on a band 7 hour of working time.
[i] Costs calculated on stated dosage and frequency; if missing, usual dose was used.
[j] Cost based on weighted average of weekday and weekend face-to-face costs for independent sector home care provided for social services.
[k] Based on median hourly earnings for all employees.
[l] Mileage was costed using NHS Terms and Conditions of Service Handbook[22]. Missing non-GP unit costs of travel were imputed using GP unit costs of travel.

Table 55 Mean resource use from an NHS and PSS perspective by category and randomised allocation

Resource Use Category: NHS and PSS (unit of measurement)	STAR care pathway		Usual Care	
	N	Mean Resource Use (95% CI)	N	Mean Resource Use (95% CI)
STAR care pathway – assessment clinic time (minutes of clinic – excluding x-ray time)	242	47.71 (45.33, 50.10)	121	0 (0,0)
STAR care pathway follow-up calls (number of calls)	224	3.07 (2.85, 3.28)	121	0 (0,0)
Inpatient stays (number of stays)	228	0.13 (0.09, 0.18)	112	0.25 (0.12, 0.38)
Subsequent outpatient visits (number of visits)	228	7.21 (6.22,8.21)	112	7.29 (5.83, 8.76)
Outpatient procedures (number of procedures)	228	0.13 (0.02, 0.23)	112	0.04 (0.00, 0.07)
Radiology visits (number of visits)	228	0.65 (0.48, 0.82)	112	0.79 (0.53, 1.05)
A&E (number of visits)[1]	226	0.14 (0.09, 0.20)	111	0.14 (0.05, 0.24)
6 months: GP contacts (practice visits, home visits, phone calls) (number of contacts) [3]	201	0.97 (0.70, 1.23)	97	0.51 (0.25, 0.76)
12 months:GP contacts (number of contacts) [3]	196	0.51 (0.31,0.71)	92	0.32(0.12,0.51)
6-months: NHS Physiotherapist/ Acupuncture/ Hydrotherapy visits (number of visits) [3]	200	1.32 (0.84,1.79)	96	1.65 (0.73, 2.58)
12 months: NHS Physiotherapist/ Acupuncture/ Hydrotherapy visits (number of visits) [3]	197	0.60 (0.17,1.02)	92	0.26 (0.06,0.46)
6 months: Other NHS community-based health service contacts[2,3] (number of contacts)	198	0.89 (0.33, 1.44)	96	0.81 (0.22, 1.40)
12 months: Other NHS community-based health service contacts (number of contacts) [3]	196	0.31 (0.04,0.58)	92	0.14 (-.01,0.30)
6 months:Home care worker NHS paid (number of visits) [3]	206	0.50 (-0.28, 1.29)	100	0.78 (-0.37, 1.92)
12 months:Home care worker NHS paid (number of visits) [3]	199	0.13 (-0.13,0.39)	92	0 (0,0)
6 months: Home changes/equipment (NHS/PSS provided only) (number of home changes/equipment) [3]	199	0.38 (0.24,0.53)	91	0.40 (0.18, 0.61)
12 months:Home changes/equipment (NHS/PSS provided only) (number of home changes/equipment) [3]	195	0.05 (0.01,0.9)	89	0.10 (0.02,0.18)
6 months: Medications (number of prescribed medications)[3]	210	1.88 (1.61,2.15)	100	1.69(1.34,2.01)
12 months: Medications (number of prescribed medications)[3]	201	1.23 (1.00,1.46)	95	1.53 (1.19,1.87)

[1] Obtained from informatic data except for patients at Birmingham and Oswestry centres, where questionnaire information was used
[2]nurse practice visits, nurse phone calls, district nurse visits, NHS 111 and other community care.
[3] If the question had not been answered, but other items within the question section had been answered then no contact or use of this resource was assumed.

Table 56 Mean resource use from a patient perspective by category and randomised allocation[1]

Resource Use Category: Patient (unit of measurement):	STAR care pathway		Usual Care	
	N	Mean Resource Use (95% CI)	N	Mean Resource Use (95% CI)
6 months: Private Physiotherapist/ Acupuncture/ Hydrotherapy visits (number of visits)	205	0.51 (0.19, 0.84)	97	0.15 (0.01, 0.30)
12 months: Private Physiotherapist/ Acupuncture/ Hydrotherapy visits (number of visits)	197	0.06 (-0.01,0.13)	92	0.12 (-0.10,0.34)
6 months: Other private community-based health service contacts (number of contacts)	205	0.28 (0.06,0.50)	97	0.18 (-0.04, 0.39)
12 months: Other private community-based health service contacts (number of contacts)	198	0.64 (-0.13,1.41)	93	0.54 (-0.05,0.16)
6 months:Prescription Charges (number of charges)	235	0.23 (0.12,0.35)	116	0.18 (0.03,0.33)
12 months: Prescription Charges (number of charges)	232	0.13 (0.05,0.21)	116	0.22 (0.06,0.39)
6 months: non prescription medication (% of participants)	196	0.39 (0.32,0.46)	87	0.39 (0.29,0.50)
12 months: non prescription medication (% of participants)	196	0.30 (0.24,0.37)	90	0.32 (0.22,0.42)
6 months:Home changes/equipment (privately purchased only) (number of home changes/equipment)	199	0.18 (0.10, 0.26)	89	0.10 (0.03, 0.17)
12 months:Home changes/equipment (privately purchased only) (number of home changes/equipment)	195	0.04 (0.01,0.07)	90	0.09 (0.01,0.16)
6 months: Home care worker privately paid (number of visits)	206	0.50 (-0.49,1.50)	100	0.00
12 months: Home care worker privately paid (number of visits)	199	0.00	83	0.00
6 months: Hours of Unpaid Leave (number of hours)	205	7.06 (1.09, 13.02)	100	12.78 (-5.61,31.17)
12 months: Hours of Unpaid Leave (number of hours)	197	2.61 (-2.54,7.76)	93	8.11 (-2.2,18.41)

[1] If the question item had not been answered, but other items within the question section had been answered then no contact or use of this resource was assumed.

Table 57 Mean costs from an NHS and PSS perspective by category and randomised allocation

Resource Use Category: NHS and PSS	STAR care pathway		Usual Care	
	N	Mean Costs £ (95% CI)	N	Mean Costs £ (95% CI)
STAR care pathway – assessment clinic (including x-rays and follow up calls)	242	183.75 (177.05, 190.47)	121	0 (0,0)
Inpatient admissions	228	494.00 (241.23,746.78)	112	1184.12 (543.01,1825.22)
Subsequent outpatient visits	228	684.46 (595.74,773.17)	112	699.70 (570.90,828.50)
Outpatient procedures	228	21.30 (3.40,39.20)	112	6.44 (-0.17,13.04)
Radiology visits	228	56.03 (38.25,73.81)	112	66.32 (43.56,89.07)
A&E[1]	226	28.70 (17.72,39.68)	111	31.79 (11.08,52.56)
6 months: GP contacts (practice visits, home visits, phone calls) [3]	201	34.23 (24.40,44.07)	97	18.23 (8.92,27.53)
12 months:GP contacts[3]	196	17.98 (11.12,24.83)	92	11.65 (4.71,18.58)
6-months: NHS Physiotherapist/ Acupuncture/ Hydrotherapy visits[3]	200	63.12 (40.13,86.11)	96	79.5 (35.26,123.74)

12 months: NHS Physiotherapist/ Acupuncture/ Hydrotherapy visits[3]	197	28.75 (8.38,49.18)	92	12.52 (2.76,22.28)
6 months: Other NHS community-based health service contacts[2,3]	198	16.45 (3.81,29.09)	96	20.64 (5.64,35.64)
12 months: Other NHS community-based health service contacts [3]	196	6.45 (0.53,12.27)	92	0.62 (-0.17,1.41)
6 months:Home care worker NHS paid [3]	205	1.92 (-1.97,5.71)	100	27.56 (-19.88,75.00)
12 months:Home care worker NHS) [3]	199	1.98 (-1.92,5.88)	91	0 (0,0)
6 months: Home changes/equipment (NHS/PSS provided only) [3]	199	285.58(40.76,530.40)	91	108.13 (20.78,195.49
12 months:Home changes/equipment (NHS/PSS provided only) [3]	195	5.98 (1.28,10.68)	89	301.85 (10.29,593.40)
6 months: Medications [3]	210	31.66 (23.48,39.84)	100	33.05 (22.30,43.80)
12 months: Medications[3]	201	21.21 (14.48,27.94)	94	26.54 (17.90,35.17)

[1] Obtained from informatic data except for patients at Birmingham and Oswestry centres, where questionnaire information was used
[2]nurse practice visits, nurse phone calls, district nurse visits, NHS 111 and other community care.
[3] If the question had not been answered, but other items within the question section had been answered then no contact or use of this resource was assumed.

Table 58 Mean utilities over time and by randomised allocation

**Time point**	**STAR care pathway**		**Usual Care**	
	**N**	**Mean utility (95% CI)**	**N**	**Mean utility (95% CI)**
Baseline	239	0.448 (0.420, 0.475)	119	0.448(0.410,0.487)
6 months	212	0.547 (0.514, 0.579)	103	0.508 (0.457, 0.558)
12 months	212	0.566 (0.532,0.600)	101	0.538 (0.489, 0.587)

Table 59 VAS scores over time and by randomised allocation

**Time point**	**STAR care pathway**		**Usual Care**	
	**N**	**Mean VAS score (95% CI)**	**N**	**Mean VAS score (95% CI)**
Baseline	242	59.01 (56.43,61.59)	121	60.24 (56.64,63.84)
6 months	214	63.49 (60.63, 66.35)	104	62.36 (58.24,66.47)
12 months	213	65.22 (62.26, 68.18)	101	58.78 (53.84, 63.73)

Table 60 One-way sensitivity analyses for the economic evaluation

	Adjusted Costs[1] (£)	Adjusted QALYs[1]	Incremental Costs	Incremental QALYs	Incremental NMB (£) at £20,000/QALY	Probability cost-effective at £20k per QALY threshold
	Mean (95% CI)	Mean (95% CI)	(95% CI)	(95% CI)	(95% CI)	
1)Altering ESP direct to indirect time to: a)1:0; NHS & PSS perspective						
STAR care pathway	1940.69 (1499.90,2381.49)	0.52 (0.50,0.54)				
Usual Care Pathway	2687.06 (2030.86, 3343.27)	0.50 (0.47,0.52)				
STAR vs usual care			-746.37 (-1539.64, 46.90)	0.03 (-0.007,0.06)	1291 (199,2384)	98.97
1b) 1:3:NHS & PSS perspective						
STAR care pathway	2187.37 (1744.53,2630.21)	0.52 (0.50,0.54)				
Usual Care Pathway	2686.41 (2030.90,3341.92)	0.50 (0.47,0.52)				
STAR vs usual care			-499.04 (-1298.46,300.37)	.0.03 (-0.007,0.06)	1038 (-58,2135)	96.83
2) Changing Health Care worker visit assumption from a) 6 to 3 months: NHS&PSS perspective						
STAR care pathway	1960.13 (1530.27,2389.98)	0.52 (0.50,0.54)				
Usual care	2672.88 (2022.45,3323.32)	0.50 (0.47,0.52)				
STAR vs UC			-712.76 (-1487.09,61.57)	0.03 (-0.008,0.06)	1244 (153,2335)	98.73
2a) 6 to 3 months: patient perspective						
STAR care pathway	371.03(151.22,590.84)	0.52 (0.50,0.54)				
Usual care	681.39 (306.29,1056.48)	0.50 (0.47,0.52)				
STAR vs usual care			-310.36 (-748.11,127.39)	0.03 (-0.008,0.06)	841 (12,1671)	97.66
2b) 6 to 1 month: NHS/PSS perspective						
STAR care pathway	1958.94 (1529.63,2388.25)	0.52 (0.50,0.54)				
Usual care	2663.61 (2013.69,3313.54)	0.50 (0.47,0.52)				

STAR vs usual care			-704.67 (-1478.17,68.82)	0.03 (-0.008,0.06)	1236 (146,2326)	98.69
2b) 6 to 1 month: patient perspective						
STAR care pathway	367.92 (148.24,587.60)	0.52 (0.50,0.54)				
Usual care	680.34 (305.48,1055.20)	0.50 (0.47,0.52)				
STAR vs usual care			-312.42 (-749.97,125.13)	0.03 (-0.008,0.06)	843 (14,1673)	97.68
3.Reducing the cost of equipment and home changes: a) by half: NHS&PSS perspective						
STAR care pathway	1822.56 (1431.25,2213.87)	0.52 (0.50,0.54)				
Usual care	2485.77 (1894.23,3077.32)	0.50 (0.47,0.52)				
STAR vs usual care			-663.21 (-1368.96,42.54)	0.03 (-0.008,0.06)	1195 (162,2228)	98.83
3a) by half: patient perspective						
STAR care pathway	288.67(120.04,457.30)	0.52 (0.50,0.54)				
Usual care	534.22 (252.01,816.42)	0.50 (0.47,0.52)				
STAR vs usual care			-245.54 (-577.06,85.97)	0.03 (-0.008,0.06)	777 (2,1551)	97.53
3b) by three quarters: NHS&PSS perspective						
STAR care pathway	1752.92 (1371.70,2134.14)	0.52 (0.50,0.54)				
Usual care	2385.52 (1810.91,2960.13)	0.50 (0.47,0.52)				
STAR vs usual care			-632.60 (-1319.39,54.20)	0.03 (-0.008,0.06)	1164 (149,2179)	98.77
3b) by three quarters: patient perspective						
STAR care pathway	245.25 (92.00,398.50)	0.52 (0.50,0.54)				
Usual care	459.88 (205.12,714.64)	0.50 (0.47,0.53)				
STAR vs usual care			-214.63 (-513.97,84.72)	0.03 (-0.008,0.06)	746 (-14,1505)	97.28
4) Assuming equipment and home changes are NHS/PSS funded if funding source not known. NHS&PSS perspective						

STAR care pathway	2020.58 (1570.68,2470.47)	0.52 (0.50,0.54)				
Usual care	2660.08(2004.64,3315.53)	0.50 (0.47,0.52)				
STAR vs usual care			-639.51 (-1431.08,152.07)	0.03 (-0.008,0.06)	1175 (86,2265)	98.27
5) Dropping "other" community based visits costs, which potentially could be double counted. NHS&PSS perspective						
STAR care pathway	1965.45 (1523.63,2407.27)	0.52 (0.50,0.54)				
Usual care	2646.40 (1995.94,3296.87)	0.50 (0.47,0.52)				
STAR vs usual care			-680.95 (-1469.96,108.05)	0.03 (-0.007,0.06)	1226 (123,2328)	98.53
6) Replacing inpatient short stay and long stay non elective costs with elective costs. NHS & PSS perspective						
STAR care pathway	1834.00 (1468.10,2199.89)	0.52 (0.50,0.54)				
Usual care	2333.47 (1802.23,2864.71)	0.50 (0.47,0.52)				
STAR vs usual care			-499.47 (-1152.22,153.28)	0.03 (-0.008,0.06)	1024 (27,2020)	97.80

[1] All variables are adjusted for site and baseline BPI subscores. Additionally QALYs were adjusted for baseline utility

STAR trial sites

Southmead Hospital, North Bristol NHS Trust

Princess Elizabeth Orthopaedic Centre, Royal Devon and Exeter NHS Trust

Llandough Hospital, Cardiff and Vale University Health Board

The Robert Jones and Agnes Hunt Orthopaedic Hospital NHS Trust

King's Mills Hospital, Sherwood Forest Hospitals NHS Trust

Wrightington Hospital, Wrightington, Wigan and Leigh NHS Trust

Leicester General Hospital, University Hospitals of Leicester NHS Trust

The Royal Orthopaedic Hospital NHS Foundation Trust

Trial Steering Committee members

Paul Ewings, NIHR Research Design Service South West, Chair.

Joy Adamson, University of Newcastle, independent member.

George Peat, Keele University, independent member.

Mark Rockett, Plymouth Hospitals NHS Trust/Plymouth University, independent member.

Lizzy Betts, patient representative.

Members of the STAR trial group

Tim Board, Wrightington, Wigan and Leigh NHS Foundation Trust

Amanda Burston, University of Bristol

Benjamin Burston, The Robert Jones and Agnes Hunt Orthopaedic Hospital

Jane Dennis, University of Bristol

Vikram Desai, Sherwood Forest Hospitals NHS Foundation Trust

Paul Dieppe, University of Exeter

Colin Esler, University Hospitals of Leicester NHS Trust

Michael Parry, The Royal Orthopaedic Hospital NHS Foundation Trust

Jonathan R.A. Phillips, Royal Devon and Exeter NHS Foundation Trust

References

1. Singh JA, Mahowald ML, Noorbaloochi S. Intraarticular botulinum toxin A for refractory painful total knee arthroplasty: a randomized controlled trial. *J Rheumatol* 2010; **37**(11): 2377-86.
2. Ma SH. Denervation therapy for intractable pain of knee joint after total knee arthroplasty. *Chinese J Tissue Eng Res* 2016; **20**(31): 4589-95.
3. Pickering G, Voute M, Macian N, Ganry H, Pereira B. Effectiveness and safety of 5% lidocaine-medicated plaster on localized neuropathic pain after knee surgery: a randomized, double-blind controlled trial. *Pain* 2019; **160**(5): 1186-95.
4. Qudsi-Sinclair S, Borrás-Rubio E, Abellan-Guillén JF, Padilla Del Rey ML, Ruiz-Merino G. A comparison of genicular nerve treatment using either radiofrequency or analgesic block with corticosteroid for pain after a total knee arthroplasty: A double-blind, randomized clinical study. *Pain practice : the official journal of World Institute of Pain* 2017; **17**(5): 578-88.
5. Pilot study of PEMF therapy in treatment of post-operative pain following total knee arthroplasty. *https://clinicaltrialsgov/show/NCT02211534* 2014.
6. Radiofrequency for chronic knee pain post-arthroplasty. *https://clinicaltrialsgov/show/NCT02931435* 2016.
7. Cannabinoids vs. placebo on persistent post-surgical pain following TKA: a pilot RCT. *https://clinicaltrialsgov/show/NCT03825965* 2019.
8. Efficacy of genicular nerve radiofrequency. *https://clinicaltrialsgov/show/NCT04100707* 2019.
9. Phenol neurolysis of genicular nerves for chronic knee pain. *https://clinicaltrialsgov/show/NCT03973177* 2019.
10. Effects of Exercise and Education in Patients With Chronic Pain After Total Knee Replacement. *https://clinicaltrialsgov/show/NCT03886259* 2019.
11. Larsen JB, Skou So T, Arendt-Nielsen L, Simonsen O, Madeleine P. Neuromuscular exercise and pain neuroscience education compared with pain neuroscience education alone in patients with chronic pain after primary total knee arthroplasty: Study protocol for the NEPNEP randomized controlled trial. *Trials* 2020; **21**(1): 218.
12. Curtis L, Burns A. Unit costs of health and social care. University of Kent, Canterbury: Personal Social Services Research Unit [Available from: https://www.pssru.ac.uk/project-pages/unit-costs/unit-costs-2020/], 2020.
13. NHS Reference Costs 2018 to 2019. Available from: https://improvement.nhs.uk/resources/national-cost-collection/. London: NHS, 2020.
14. Curtis L. Unit costs of health and social care. University of Kent, Canterbury: Personal Social Services Research Unit [Available from: https://www.pssru.ac.uk/project-pages/unit-costs/unit-costs-2013/, 2013.
15. Curtis L, Burns A. Unit costs of health and social care. University of Kent, Canterbury: Personal Social Services Research Unit [Available from: https://www.pssru.ac.uk/project-pages/unit-costs/unit-costs-2015/], 2015.
16. Turner J, O'Cathain A, Knowles E, Nicholl J, Tosh J, Sampson F. Evaluation of NHS 111 pilot sites. Final Report. University of Sheffield: Medical Care Research Unit; [Available from: https://www.sheffield.ac.uk/polopoly_fs/1.227404!/file/NHS_111_final_report_August_2012.pdf]. 2012.
17. British National Formulary. London: NICE [Available from: https://bnf.nice.org.uk/] accessed December 2020., 2020.
18. Am I entitled to free prescriptions? London: NHS [Available from: https://www.nhs.uk/using-the-nhs/help-with-health-costs/get-help-with-prescription-costs/], 2020.
19. Coalville. Leicestershire: NRS Healthcare; Available from: https://www.nrshealthcare.co.uk/] accessed November 2020. 2020.
20. Curtis L, Burns A. Unit costs of health and social care. University of Kent, Canterbury: Personal Social Services Research Unit [Available from: https://www.pssru.ac.uk/project-pages/unit-costs/unit-costs-2019/], 2019.
21. Annual Survey of Hours and Earnings (ASHE). Newport, South Wales: Office for National Statistics; 2020 [Available from: https://www.ons.gov.uk/employmentandlabourmarket/peopleinwork/earningsandworkinghours/datasets/allemployeesashetable1]. 2020.
22. NHS Terms and Conditions of Service Handbook. London: The NHS Staff Council; [Available from: https://www.nhsemployers.org/employershandbook/tchandbook/afc_tc_of_service_handbook_fb.pdf]. 2019.
